# Investigating Novel Therapeutic Approaches for Idiopathic Short Stature: Targeting siRNA and Growth Hormone Delivery to the Growth Plate Using Exosome Nanoparticles

**DOI:** 10.1002/advs.202309559

**Published:** 2024-04-19

**Authors:** Jinghong Yuan, Yameng Wang, Yanzhe Huang, Shengqin Li, Xiaowen Zhang, Zhiwen Wu, Wenrui Zhao, Junchao Zhu, Junqiu Zhang, Guowen Huang, Peng Yu, Xigao Cheng, Xinhui Wang, Xijuan Liu, Jingyu Jia

**Affiliations:** ^1^ Department of Orthopaedics The Second Affiliated Hospital of Nanchang University Nanchang 330006 P. R. China; ^2^ Institute of Orthopaedics of Jiangxi Province Nanchang 330006 P. R. China; ^3^ Department of Pediatrics The Second Affiliated Hospital of Nanchang University Nanchang 330006 P. R. China; ^4^ Department of Endocrinology and Metabolism The Second Affiliated Hospital of Nanchang University Nanchang 330006 P. R. China; ^5^ Division of Gastrointestinal and Oncologic Surgery Department of Surgery Massachusetts General Hospital Harvard Medical School Boston MA 02114 USA

**Keywords:** bone growth, exosome, growth plate, idiopathic short stature, long noncoding RNA

## Abstract

Idiopathic short stature (ISS) is a common childhood condition with largely unknown underlying causes. Recent research highlights the role of circulating exosomes in the pathogenesis of various disorders, but their connection to ISS remains unexplored. In the experiments, human chondrocytes are cocultured with plasma exosomes from ISS patients, leading to impaired chondrocyte growth and bone formation. Elevated levels of a specific long non‐coding RNA (lncRNA), ISSRL, are identified as a distinguishing factor in ISS, boasting high specificity and sensitivity. Silencing ISSRL in ISS plasma exosomes reverses the inhibition of chondrocyte proliferation and bone formation. Conversely, overexpression of ISSRL in chondrocytes impedes their growth and bone formation, revealing its mechanism of action through the miR‐877‐3p/GZMB axis. Subsequently, exosomes (CT‐Exo‐siISSRL‐oeGH) with precise cartilage‐targeting abilities are engineered, loaded with customized siRNA for ISSRL and growth hormone. This innovative approach offers a therapeutic strategy to address ISS by rectifying abnormal non‐coding RNA expression in growth plate cartilage and delivering growth hormone with precision to promote bone growth. This research provides valuable insights into ISS diagnosis and treatment, highlighting the potential of engineered exosomes.

## Graphical abstract

Plasma exosomes from idiopathic short stature (ISS) individuals hinder chondrocyte growth, driving ISS development through the ISSRL/miR‐877‐3P/Granzyme B (GZMB) axis. The innovative approach utilizes engineered exosomes (CT‐Exo‐siISSRL‐oeGH) for precise siRNA and growth hormone delivery, mitigating abnormal RNA effects on growth plate development while enhancing bone growth. This research provided a poineering strategy for addressing ISS.

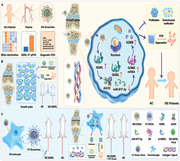

## 1 Introduction

Short stature in children and adolescents often gives rise to emotional challenges, including feelings of inferiority, anxiety, and depression.^[^ ^1^, ^2^ ^]^ Moreover, it can have lasting impacts on adult life, influencing career choices and even the selection of a life partner. Idiopathic short stature (ISS) accounts for a significant majority, approximately 80%, of all cases of short stature.^[^ ^3^ ^]^ To be diagnosed with ISS, individuals must undergo thorough evaluations to exclude systemic diseases, nutritional deficiencies, psychiatric disorders, chromosomal abnormalities, and overt hormonal imbalances. Additionally, their height must be more than 2 standard deviations (SDs) below the mean height for their age and sex, with no evidence of underlying systemic, chromosomal, nutritional, psychological, or hormonal disorders.^[^ ^4^, ^5^ ^]^ From the definition of ISS, it can be seen that the etiology of ISS remains unclear.

Advanced genetic screening techniques have been employed to unravel the intricate mechanisms underlying ISS. These investigations have revealed mutation rates ranging from 1% to 6% for the genes associated with this condition. Specifically, ACAN demonstrates a mutation rate of 1.4%,^[^ ^6^ ^]^ SHOX ranges from 2% to 4%,^[^ ^7^ ^]^ while GHR and NPR2 exhibit mutation rates of approximately 5% and 6%,^[^ ^8^ ^]^ respectively. Remarkably, only a limited subset of ISS cases exhibit mutations in these pivotal genes.^[^ ^9^ ^]^ These findings highlight the complexity of idiopathic short stature and emphasize the urgent need for further research to comprehensively understand its underlying pathophysiology.

It is widely established that growth plate cartilage is responsible for bone longitudinal elongation and that circulating chemicals such as hormones and growth factors influence growth plate cartilage production.^[^ ^10^, ^11^ ^]^ Alongside hormones and growth factors, recent studies have unveiled that plasma contains a high concentration of exosomes.^[^ ^12^ ^]^ Exosomes, being membrane‐bound vesicles, typically range in size from 30 to 150 nm.^[^ ^13^ ^]^ They are secreted by various cell types and facilitate cell‐to‐cell communication by transferring proteins, microRNAs, long noncoding RNAs, and circRNAs. Notably, multiple studies have demonstrated the significant role of circulating exosomes in intercellular communication, information transfer, gene regulation, and the progression of various diseases through their cargo of proteins, microRNAs, long noncoding RNAs (lncRNAs), and circRNAs. For instance, the research of Jin et al.^[^ ^14^ ^]^ revealed that exosomes carrying microRNA‐26a‐5p from human bone mesenchymal stem cells mitigated synovial fibroblast injury and alleviated osteoarthritis by regulating PTGS2. Yan et al.^[^ ^15^ ^]^ found that overexpression of the lncRNA H19 within exosomes from umbilical cord mesenchymal stem cells enhanced osteochondral activity by upregulating lncRNA H19. However, despite these advances, the role of plasma exosomal lncRNAs in ISS pathogenesis remains unexplored.

Due to the unclear etiology of ISS, there is currently a lack of effective treatment options. Recombinant human growth hormone (rhGH) has been utilized to treat ISS since 2003. However, unlike individuals with growth hormone deficiency‐related short stature, children with ISS do not exhibit a lack of growth hormone. The efficacy of rhGH treatment varies significantly across studies, with limited benefits, uncertain outcomes, and substantial economic burden.^[^ ^6^, ^7^, ^8^, ^9^ ^]^ Chung et al.^[^ ^9^ ^]^ reported that the 6 month mean growth velocity of individuals having ISS increased from 5.63 to 10.08 cm per year after rhGH treatment in comparison to that of the untreated group from 4.94 to 5.92 cm per year. Moreover, Kim et al.^[^ ^6^ ^]^ observed in the phase III randomized trial that ISS patients treated with rhGH showed an increasing annual height velocity (10.68 ± 1.95 cm per year) compared with the control group patients (5.72 ± 1.72 cm per year). However, Van Balen et al.^[^ ^7^ ^]^ documented that although following rhGH treatment the height SDS in ISS patients underwent a significant improvement, bone maturation manifested a significant acceleration in the rhGH treatment group. Consequently, no notable difference was observed in adult height among the rhGH therapy group and the control group (169.7 ± 4.2 and 168.8 ± 3.8, respectively).

Notably, concerns about the long‐term safety of rhGH treatment, especially at high doses, have emerged. Carel et al.^[^ ^10^ ^]^ reported an increased mortality rate in 871 ISS patients treated with rhGH, attributed to subarachnoid or intracerebral hemorrhage, cardiovascular diseases, and bone and cartilage cancers. Subsequently, Ying et al.^[^ ^11^ ^]^ identified a 21.5% chance of hyperglycemia and a 17% chance of hyperinsulinemia after rhGH treatment in ISS patients. Considering these potential risks, targeted delivery of rhGH to the growth plate may be a promising approach to mitigate complications associated with rhGH therapy for ISS. However, it's important to note that no research is currently available on the targeted delivery of growth hormone to the growth plate.

The current study has the primary objective of elucidating the distinct expression profile of lncRNAs within plasma exosomes of individuals affected by ISS. Additionally, this research aims to uncover the underlying biological roles of the identified candidate lncRNAs in the pathogenesis of ISS, employing a combination of in vivo and in vitro experiments. Simultaneously, we are employing genetic engineering techniques to construct exosome nanoparticles that carry siRNA‐lncRNA and growth hormone (GH), with a specific cartilage‐targeting effect (CT‐Exo‐siISSRL‐oeGH). For the first time, we propose the targeted delivery of siRNA to mitigate the inhibitory effects on growth plate development caused by abnormally expressed lncRNAs, while also delivering GH to restore the growth and development of stunted rats. This innovative approach holds significant promise as a potential therapeutic avenue for effectively addressing the treatment of ISS.

## 2 Results

### 2.1 Isolation and Identification of Plasma Exosomes

**Scheme 1:**
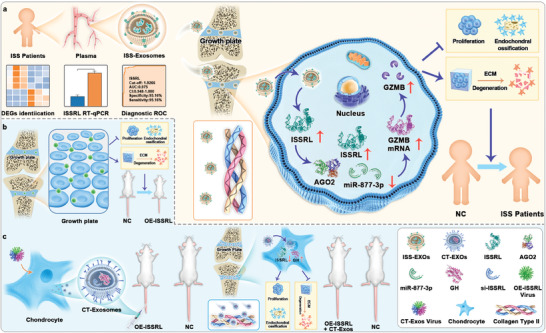
The plasma exosomes derived from ISS children exhibited inhibitory effects on both chondrocyte proliferation and endochondral ossification, while concurrently promoting extracellular matrix degradation, thereby contributing to the development of ISS. This phenomenon can be attributed to the intricate ISSRL/miR‐877‐3P/GZMB axis. In an experimental model involving immature rats, we induced the overexpression of ISSRL through the administration of lentiviral vectors via tail vein injection, which resulted in the manifestation of a short stature phenotype. Subsequently, we engineered exosomes, specifically designated as CT‐Exo‐siISSRL‐oeGH, with a refined capacity for precise targeting of the growth plate cartilage, loaded with customized siRNA for ISSRL and growth hormone. The current study introduces a novel therapeutic strategy for addressing ISS, wherein extracellular vesicle targeting technology is employed to deliver siRNA, thereby mitigating the inhibitory effect of abnormally expressed non‐coding RNA on growth plate cartilage development, while precisely delivering GH to promote bone growth and correct the short stature phenotype.

**Figure 1:**
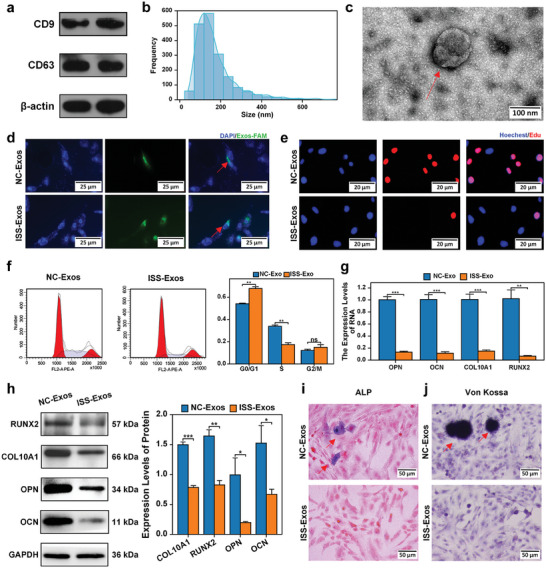
Plasma exosomes of ISS children suppressed the proliferation and bone formation of normal human chondrocytes in vitro. a) Western blot analysis confirmed the presence of exosome markers, CD63 and CD9, in the extracted samples. b) Nano‐Sight analysis verified that the size of the extracted particles was approximately 100 nm. c) The morphology of the extracted material was further examined using a transmission electron microscope. d) The uptake of FAM‐labeled exosomes (green fluorescence) by chondrocytes (blue fluorescence) was successfully validated. e) EdU staining demonstrated that human chondrocyte proliferation was suppressed following co‐culture with ISS plasma exosomes. f) Flow cytometric analysis revealed a cell cycle arrest in the G0/G1 phase. g,h) Western blot and RT‐qPCR analyses identified a significant downregulation of marker genes associated with chondrocyte differentiation (COL10A1 and RUNX2) and osteogenic genes (OPN and OCN) after human chondrocytes were cocultured with ISS plasma exosomes. i, j) The activity of ALP was reduced, and von Kossa staining revealed a decrease in mineralization after the co‐culture of human chondrocytes with ISS plasma exosomes. The data are presented as the mean±SD. *n* = 3. Two groups were compared using T‐test, ^**^ *P* < 0.01, ^***^ *P* < 0.001 versus control.

Graphical abstract is shown in **Scheme** **1**. The extraction samples showed positive expression of CD63 and CD9 (markers of exosomes) via WB evaluation in ISS children and normal children (**Figure** **1**a). Nanosight analysis verified that the size of extraction is less than 100 nm (Figure 1b). The morphology of the extraction was further examined via transmission electron microscope (Figure 1c). The authors observed that the samples of extraction showed the typical characteristics of exosomes.

### 2.2 Plasma Exosomes of ISS Children Suppressed the Proliferation and Bone Formation of Normal Human Chondrocytes In Vitro

Human chondrocytes were cocultured with ISS and normal children's plasma exosomes (Figure 1d). Human chondrocyte proliferation was decreased in the group of ISS exosomes (ISS‐Exos), according to EdU (Figure 1e). Meanwhile, the outcomes of flow cytometric analysis revealed that the number of G0/G1‐phase cells in the ISS‐Exos group was significantly high when compared with the NC exosomes group (NC‐Exos). In addition, a relatively small number of cells were observed in the S and G2/M phases, showing that G1 cell cycle arrest is more noticeable in the ISS exosomes category in comparison to the NC category (Figure 1f).

To investigate endochondral ossification, the COL10A1, RUNX2, OPN, OCN, Von Kossa staining, and alkaline phosphatase (ALP) were measured in the ISS‐Exos and NC‐Exos groups. The extent of the expression of COL10A1 and RUNX2 was substantially lower in the ISS‐Exos category than in the NC‐Exos group, according to the results of a western blot analysis and RT‐qPCR. This indicates that chondrocyte hypertrophy has been inhibited (Figure 1g,h). The expressions of the osteogenic genes (OPN and OCN) were lower in the ISS‐Exos group in comparison to the NC‐Exos group (Figure 1g,h). In the meantime, ALP activity has diminished (Figure 1i). Furthermore, Von Kossa staining revealed less mineralization (Figure 1j). The findings showed that ISS children's plasma exosomes inhibited the proliferation and bone formation of normal human chondrocytes.

### 2.3 ISSRL Overexpression Was Identified via lncRNA Microarray and RT‐qPCR, and ISSRL Showed High Diagnostic Efficiency in ISS Children

**Figure 2:**
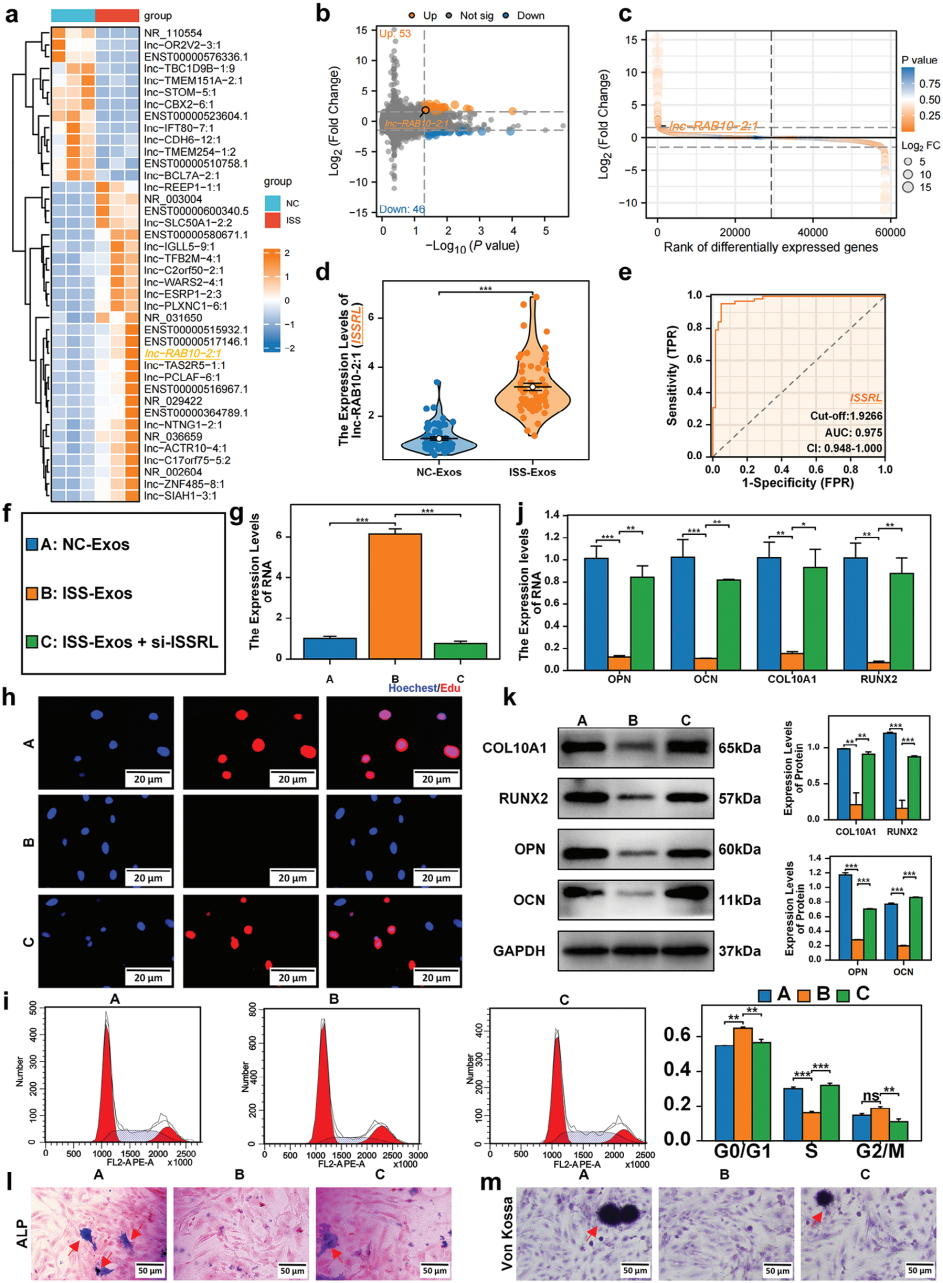
The inhibition of proliferation and bone formation in normal human chondrocytes, induced by ISS exosomes, demonstrated a noteworthy improvement upon the downregulation of lnc‐RAB10‐2:1 (ISSRL). a) The top 40 differential expression DELs were shown in a heatmap. b) The volcano plot depicts a total of 99 differentially expressed DELs, comprising 53 upregulation and 46 downregulation. Upregulated DELs are represented in orange, while down‐regulated DELs are indicated in blue. c) A rank plot of differential expression of all lncRNAs in the microarray. d) The RT‐qPCR revealed that ISSRL expression was significantly upregulated in plasma exosomes of ISS. e) An AUC of 0.975 (95% CI, 0.948–1.000) was observed. ISSRL, with the best cutoff point of >1.9266, had a specificity of 95.16% and a sensitivity of 95.16% in discriminating children with ISS from normal control children. f) Experimental Grouping: Group A (Blue): Chondrocytes co‐cultured with NC‐Exos. Group B (Orange): Chondrocytes cocultured with ISS‐Exos. Group C (Green): Chondrocytes co‐cultured with ISS‐Exos, with simultaneous silencing of ISSRL expression. g) The expression of ISSRL was increased in group B compared with group A. si‐ISSRL significantly silenced ISSRL expression after ISS‐Exos cocultured with chondrocyte. h) EdU showed that the inhibition of the chondrocyte proliferation was significantly improved after ISSRL was silenced. i) Flow cytometric analysis revealed that the cell cycle was restored to the G2/M phase after ISSRL was down‐regulated. j,k) RT‐qPCR and western blot observed the expression of marker genes of chondrocyte hypertrophy differentiation (COL10A1 and RUNX2) and the osteogenic genes (OCN and OPN) were also significantly improved after ISSRL was down‐regulated. l and m) The activity of ALP and von Kossa staining was improved after ISSRL was reduced. The data are presented as the mean±SD. *n* = 3. Three groups were compared using ANOVA followed by Tukey's test. **P < 0.01, ***P < 0.001 versus control.

Exosomes from children suffering from ISS and age/gender‐matched control people (*n* = 3 vs 3) were analyzed using lncRNA microarray (**Figure** **2**a–c). In the ISS group, there were 99 DELs, including 53 upregulated and 46 downregulated lncRNAs (*P*‐value < 0.05, |log_2_(Fold Change)| ≥ 1.5) (Figure 2b). The top 40 DELs were shown in a heatmap (Figure 2a). Because DELs are capable of regulating the expression levels of the respective target genes, GO and KEGG analyses were employed to further enrich the 99 DELs that were differentially expressed (Figure S1, Supporting Information). The greatest significant difference was found in lncRNA RAB10‐2:1 (ISSRL, ISS Related LncRNA) (Figure 2d). Subsequently, we analyzed the diagnostic efficiency of ISSRL in discriminating ISS children from normal control individuals. The AUC of 0.975 (95%CI, 0.984–1.000) was identified in the present study (Figure 2e). ISSRL, with the best cutoff point of > 1.9266, presented a specificity of 95.16% and a sensitivity of 95.16% (Figure 2e).

### 2.4 ISSRL Suppressed the Proliferation and Bone Formation of Normal Human Chondrocytes

**Figure 3:**
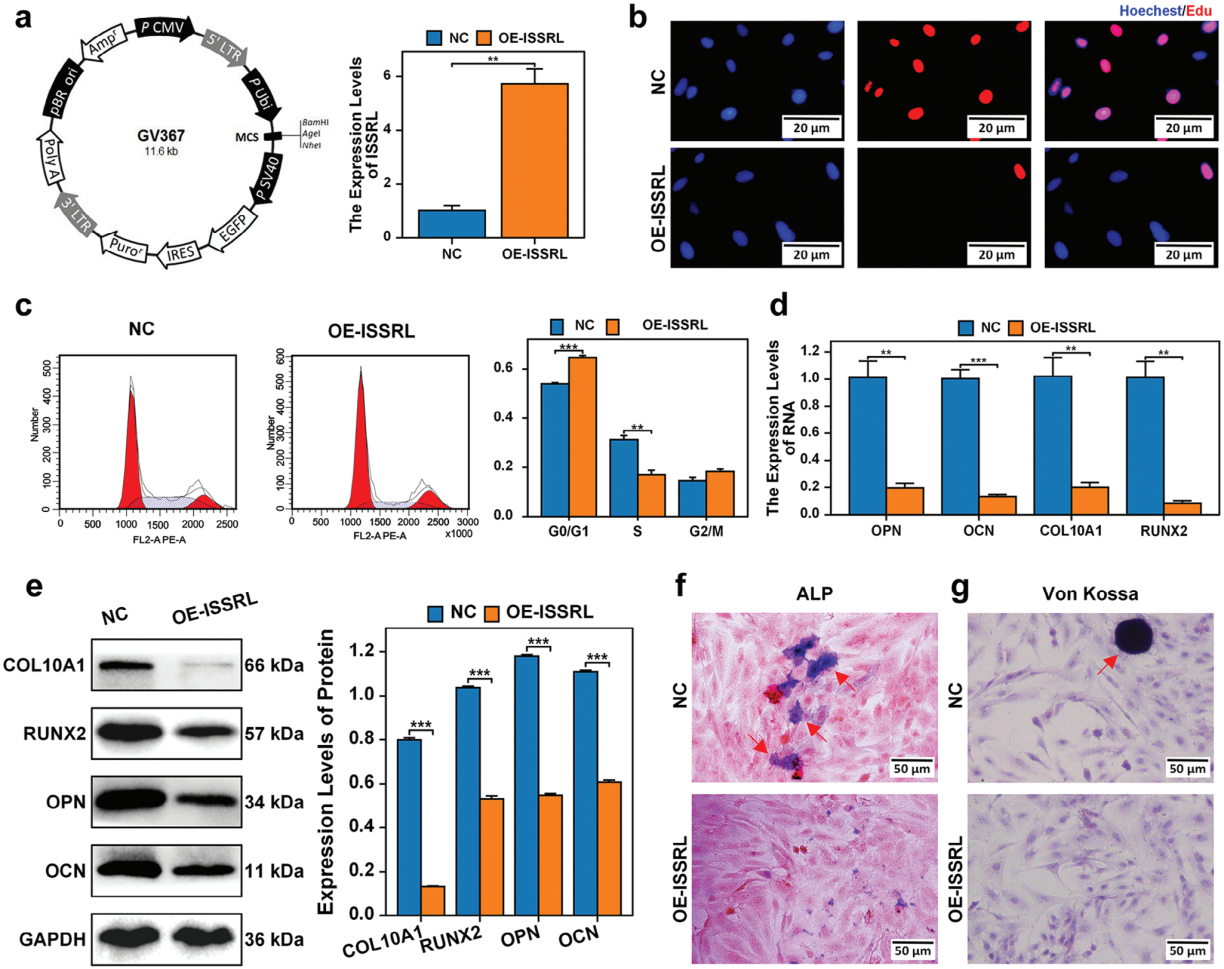
ISSRL overexpression suppressed the proliferation and bone formation of normal human chondrocytes. a) ISSRL was overexpressed in human chondrocytes using pHBLV‐ISSRL vector. b) EdU showed that upregulating ISSRL inhibited the proliferation of human chondrocytes. c) Flow cytometric analysis revealed that the cell cycle was arrested in the G0/G1 phase after the overexpression of ISSRL. d,e) RT‐qPCR and western blot identified the marker genes of chondrocyte hypertrophy differentiation (COL10A1 and RUNX2) and the osteogenic gene (OPN and OCN) showed obvious downregulation after ISSRL was up‐regulated. f,g) The activity of ALP was reduced, and von Kossa staining revealed a decrease in mineralization after ISSRL was overexpressed. The data are presented as the mean±SD. *n* = 3. Two groups were compared using T‐test. *P < 0.05, **P < 0.01, ***P < 0.001 versus control. OPN, osteopontin; OCN, osteocalcin; RUNX2, runt‐related transcription factor 2; ALP, alkaline phosphatase.

Following the coculturing of normal human chondrocytes with plasma exosomes derived from ISS children, we undertook the downregulation of ISSRL expression within the ISS plasma exosomes. The outcome of this intervention revealed a marked enhancement in the alleviation of both proliferation and bone formation inhibition within normal human chondrocytes. This improvement carried significant statistical significance (*P* < 0.05), as depicted in Figure 2f–m. These findings strongly suggested that the inhibitory effects on the proliferation and bone formation of normal human chondrocytes are attributed to the presence of ISSRL in ISS plasma exosomes. Subsequently, we proceeded to investigate the role of ISSRL in chondrocyte physiology by introducing its overexpression in human chondrocytes using pHBLV‐ISSRL vector (OE‐ISSRL, **Figure** **3**a). The outcomes of this experiment are presented below:

As illustrated in Figure 3b, the results from the EdU assay indicated that elevating ISSRL levels led to a reduction in the growth of human chondrocytes. Subsequent flow cytometry analysis revealed a diverse distribution of OE‐ISSRL chondrocytes across different cell cycle stages. Notably, the population of cells in the G0/G1 phase was significantly elevated within the OE‐ISSRL group when compared to the NC group (Figure 3c). Conversely, fewer cells within the OE‐ISSRL group were in the S and G2/M phases in comparison to the NC group, highlighting a more pronounced G1 cell cycle arrest effect within the OE‐ISSRL group (Figure 3c).

To evaluate the impact of ISSRL on endochondral ossification and chondrocyte hypertrophy, the expressions of key markers such as COL10A1, RUNX2, OCN, and OPN, Von Kossa staining, and ALP were measured after ISSRL overexpression. The RT‐qPCR and western blot analysis results, as demonstrated in Figure 3d,e, indicated a significant reduction in the expression levels of COL10A1 and RUNX2 following the overexpression of ISSRL. This downregulation suggested an inhibitory effect on chondrocyte hypertrophy. Additionally, the overexpression of ISSRL led to a decrease in the expressions of osteogenic genes (OCN and OPN), as observed through RT‐qPCR and western blot analysis (Figure 3d,e). Concurrently, ALP activity was diminished (Figure 3f). Moreover, the results of Von Kossa staining indicated reduced mineralization (Figure 3g). The comprehensive findings are depicted in Figure 3a–g collectively affirm that the overexpression of ISSRL significantly mitigates chondrocyte hypertrophy, endochondral ossification, and chondrocyte proliferation, underscoring the pivotal role of this lncRNA in these processes.

### 2.5 ISSRL Decreased the Expression of miR‐877‐3p and Upregulated GZMB Expression via AGO2 In Vitro

**Figure 4:**
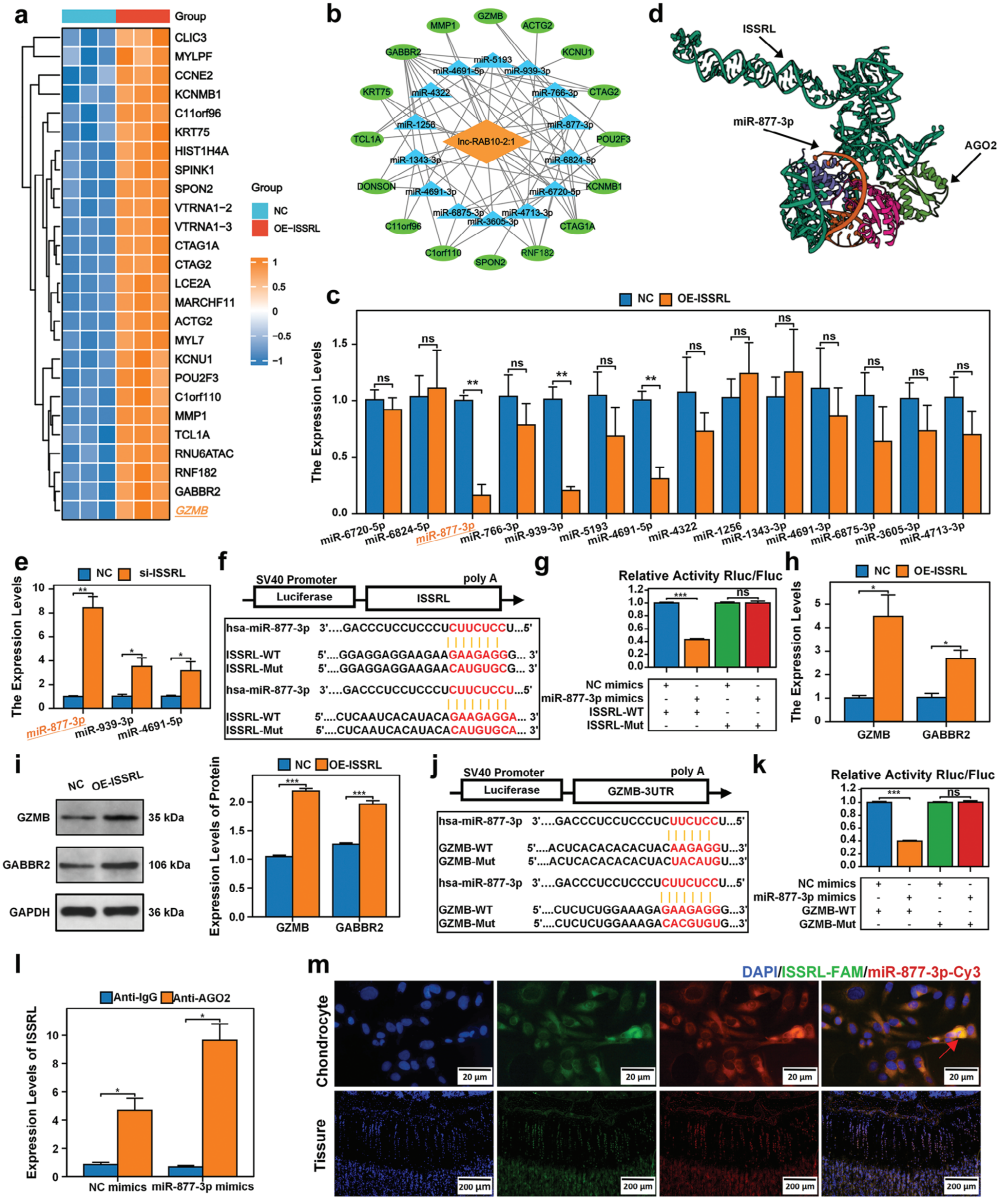
ISSRL downregulated miR‐877‐3p expression and upregulated GZMB expression via AGO2. a) A heatmap shows the 26 up‐regulated DEGs after ISSRL was up‐regulated. b) A ceRNA network of ISSRL: ISSRL is represented in orange, DEGs are depicted in green, and miRNAs are shown in blue. c) The expression of miR‐4691‐5p, miR‐877‐3p, and miR‐939‐3p exhibited a significant decrease in human chondrocytes following the overexpression of ISSRL. d) The molecular docking prediction results indicate that AGO2 can combine with ISSRL and miR‐877‐3p. e) Only 2 candidate miRNAs displayed upregulation after the downregulation of ISSRL. Meanwhile, miR‐877‐3p presented the most significant difference. f,g) The luciferase activities were recovered following co‐transfection with their mutant fragments. This suggests that miR‐877‐3p is a target of ISSRL. h,i) RT‐qPCR and western blot demonstrated that GZMB and GABBR2 were overexpressed after the upregulation of ISSRL. Meanwhile, GZMB manifested the largest significant difference after the upregulation of ISSRL. j,k) The luciferase activities were recovered following co‐transfection with their mutant fragments. This suggests that GZMB is a target of miR‐877‐3p. l) A RIP assay was performed using anti‐normal IgG or anti‐Ago2 in chondrocyte lysates. ISSRL was significantly overexpressed in AGO2‐induced immune complexes compared with IgG, and miR‐877‐3p mimics can further promote the expression of ISSRL in AGO2‐induced immune complexes. m) In situ hybridization showed ISSRL and miR‐877‐3p were colocalized in the cytoplasm of the chondrocyte and SD rat femoral growth plates. The data are presented as the mean±SD. *n* = 3. Two groups were compared using T‐test or four groups were compared using ANOVA followed by Tukey's test. *P < 0.05, **P < 0.01, ***P < 0.001 versus control.

Following the overexpression of ISSRL, mRNA high‐throughput sequencing was employed to investigate differentially expressed mRNAs (DEGs), aiming to identify potential target genes of ISSRL. Out of the identified mRNAs, a total of 68 were observed to have differential expression. Among them, 26 exhibited upregulation, while the remaining 42 displayed downregulation, with statistical significance (*P*‐value < 0.05) and a substantial |log2(Fold Change)| exceeding 3.0. The heatmap in **Figure** **4**a illustrates the expression patterns of the 26 upregulated differentially expressed genes (DEGs). GO enrichment analyses were employed for further enriching the 68 DEGs that were differentially expressed, DEGs not significantly enriched in the KEGG signaling pathway (Figure S2, Supporting Information). Subsequently, ceRNA network analysis was utilized to predict potential downstream target genes of ISSRL. This analysis showed that 14 target miRNAs and 16 DEGs were the potential target genes of ISSRL (Figure 4b). Subsequent RT‐qPCR analyses revealed that among the identified candidate miRNAs, the expression levels of three miRNAs were downregulated in human chondrocytes following the upregulation of ISSRL. Statistical analysis highlighted a negative correlation between these three candidate miRNAs (miR‐877‐3p, miR‐939‐3p, and miR‐4691‐5p) and ISSRL (Figure 4c). The molecular docking prediction results indicate that AGO2 can combine with ISSRL and miR‐877‐3p (Figure 4d). In a separate experiment where the expression of ISSRL was downregulated, only two candidate miRNAs displayed upregulation (Figure 4e). Based on the observed differences, miR‐877‐3p was selected as a candidate miRNA for further investigation. The authors conducted co‐transfection experiments involving wild‐type ISSRL (ISSRL‐WT)/miR‐877‐3p mimics with their respective mutant fragments to assess the interaction. The results revealed that co‐transfection led to decreased luciferase reporter activities in chondrocytes, which were restored upon co‐transfection with the Mut‐ISSRL (Figure 4f,g). These findings provided evidence that miR‐877‐3p is likely targeted by ISSRL in individuals with ISS conditions.

By intersecting the upregulated differentially expressed mRNAs with the predicted target mRNAs of miR‐877‐3p, two candidate genes, namely Granzyme B (GZMB) and gamma‐aminobutyric acid type B receptor subunit 2 (GABBR2), were selected for further evaluation through qPCR and western blot. Among these, GZMB exhibited the most significant and consistent differences following ISSRL modulation (Figure 4h,i). In 293T cells cotransfected with GZMB‐WT/miR‐877‐3p mimics, luciferase reporter assays revealed a decrease in activity, which was subsequently restored upon cotransfection with GZMB‐Mut and miR‐877‐3p mimics (Figure 4j,k). Previous research suggested that GZMB can mediate the degradation of cartilage proteoglycans. As anticipated, ISSRL overexpression resulted in the downregulation of proteoglycan and COL2A1 expression (Figure S3, Supporting Information).

RIP assays with anti‐AGO2 and anti‐IgG revealed that miR‐877‐3p overexpression increased the adsorption of ISSRL on anti‐AGO2 instead of anti‐IgG (Figure 4l). Furthermore, in‐situ hybridization analysis unveiled that ISSRL was primarily localized within the cytoplasm of chondrocytes (Figure 4m). This observation further suggested a potential mechanism where ISSRL might exert its biological functions by acting as a sponge for miRNAs. The in‐situ hybridization results confirmed the co‐localization of ISSRL and miR‐877‐3p in human chondrocytes, with a predominant co‐localization observed within the proliferative region of SD rat femoral growth plates (Figure 4m).

### 2.6 ISSRL Suppressed the Proliferation and Bone Formation of Normal Human Chondrocytes via miR‐877‐3p/ GZMB In Vitro

**Figure 5:**
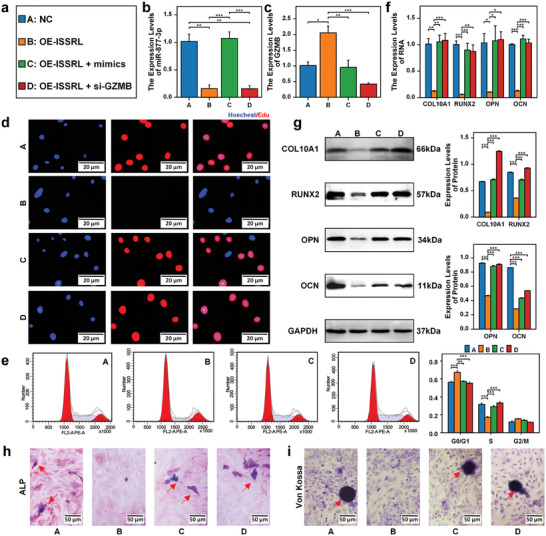
ISSRL suppressed the proliferation and bone formation of normal human chondrocytes via miR‐877‐3p/ GZMB in vitro. a) Experimental grouping: Group A (Blue): Normal chondrocytes (NC). Group B (Orange): Chondrocytes with ISSRL overexpression (OE‐ISSRL). Group C (Green): ISSRL‐overexpressed chondrocytes with increased miR‐877‐3p expression using miR‐877‐3p mimics. Group D (Red): ISSRL‐overexpressed chondrocytes with silenced GZMB expression using si‐GZMB. b) The expression of miR‐877‐3p was decreased after overexpression of ISSRL, and miR‐877‐3p mimics restored miR‐877‐3p expression. c) The expression of GZMB was increased after overexpression of ISSRL, and si‐GZMB restored GZMB expression. d) EdU showed that in the OE‐ISSRL group, chondrocyte proliferation was diminished, but this trend was reversed in the OE‐ISSRL + miR‐877‐3p mimic group and OE‐ISSRL + si‐GZMB group. e) Flow cytometric analysis revealed that the cell cycle was arrested in the G0/G1 phase after the overexpression of ISSRL, while up‐regulated miR‐877‐3p and silencing GZMB expression caused the cell cycle in the G2/M phase. f) The RT‐qPCR results revealed that mRNA expression levels of COL10A1, RUNX2, OPN, and OCN were reduced in the OE‐ISSRL group. However, no significant differences were observed between the OE‐ISSRL + miR‐877‐3p mimic group or OE‐ISSRL + si‐GZMB group and the NC group. g) The western blot outcomes indicated that protein expression levels of COL10A1, RUNX2, OPN, and OCN were reduced in the OE‐ISSRL group, but no significant differences were observed between the OE‐ISSRL + miR‐877‐3p mimic group or OE‐ISSRL + si‐GZMB group and the NC group. h) ALP activity declined in the OE‐ISSRL group but was restored in both the OE‐ISSRL + miR‐877‐3p mimic group and the OE‐ISSRL + si‐GZMB group. i) Von Kossa staining indicated reduced mineralization in the OE‐ISSRL group, whereas mineralization was regained in the OE‐ISSRL + miR‐877‐3p mimic group and OE‐ISSRL + si‐GZMB group. The data are presented as the mean±SD. *n* = 3. Two groups were compared using T‐test or four groups were compared using ANOVA followed by Tukey's test. *P < 0.05, **P < 0.01, ***P < 0.001 versus control.

Subsequent experiments involving the overexpression of ISSRL demonstrated its inhibitory effect on endochondral ossification, hypertrophy, and chondrocyte growth. Conversely, elevated expression of miR‐877‐3p and knockdown of GZMB counteracted these effects, as illustrated in **Figure** **5**a–i. The RT‐qPCR results revealed that miR‐877‐3p expression was reduced, and GZMB was up‐regulated in the OE‐ISSRL group. In contrast, miR‐877‐3p expression was increased in the OE‐ISSRL + miR‐877‐3p mimic group, and GZMB expression was down‐regulated in the OE‐ISSRL + si‐GZMB group, as depicted in Figure 5b,c. For instance, in the OE‐ISSRL group, chondrocyte proliferation was diminished, but this trend was reversed in the OE‐ISSRL + miR‐877‐3p mimic group and OE‐ISSRL + si‐GZMB group, as shown in Figure 5d,e. While mRNA and protein expression levels of COL10A1, RUNX2, OPN, and OCN were reduced in the OE‐ISSRL group, no significant differences were observed between the OE‐ISSRL + miR‐877‐3p mimic group or OE‐ISSRL + si‐GZMB group and the NC group, as presented in Figure 5f,g.

ALP activity declined in the OE‐ISSRL group but recovered in the OE‐ISSRL + miR‐877‐3p mimic group and OE‐ISSRL + si‐GZMB group, as depicted in Figure 5h. Similarly, Von Kossa staining indicated reduced mineralization in the OE‐ISSRL group, whereas mineralization was regained in the OE‐ISSRL + miR‐877‐3p mimic group and OE‐ISSRL + si‐GZMB group, consistent with ALP activity, as displayed in Figure 5i. These comprehensive findings demonstrate that the overexpression of ISSRL suppresses endochondral ossification, hypertrophy, and chondrocyte proliferation through the regulation of the miR‐877‐3p/GZMB axis in vitro.

### 2.7 Growth Plate Cartilage‐Targeting Property of CT‐Exo‐siISSRL‐oeGH

**Figure 6:**
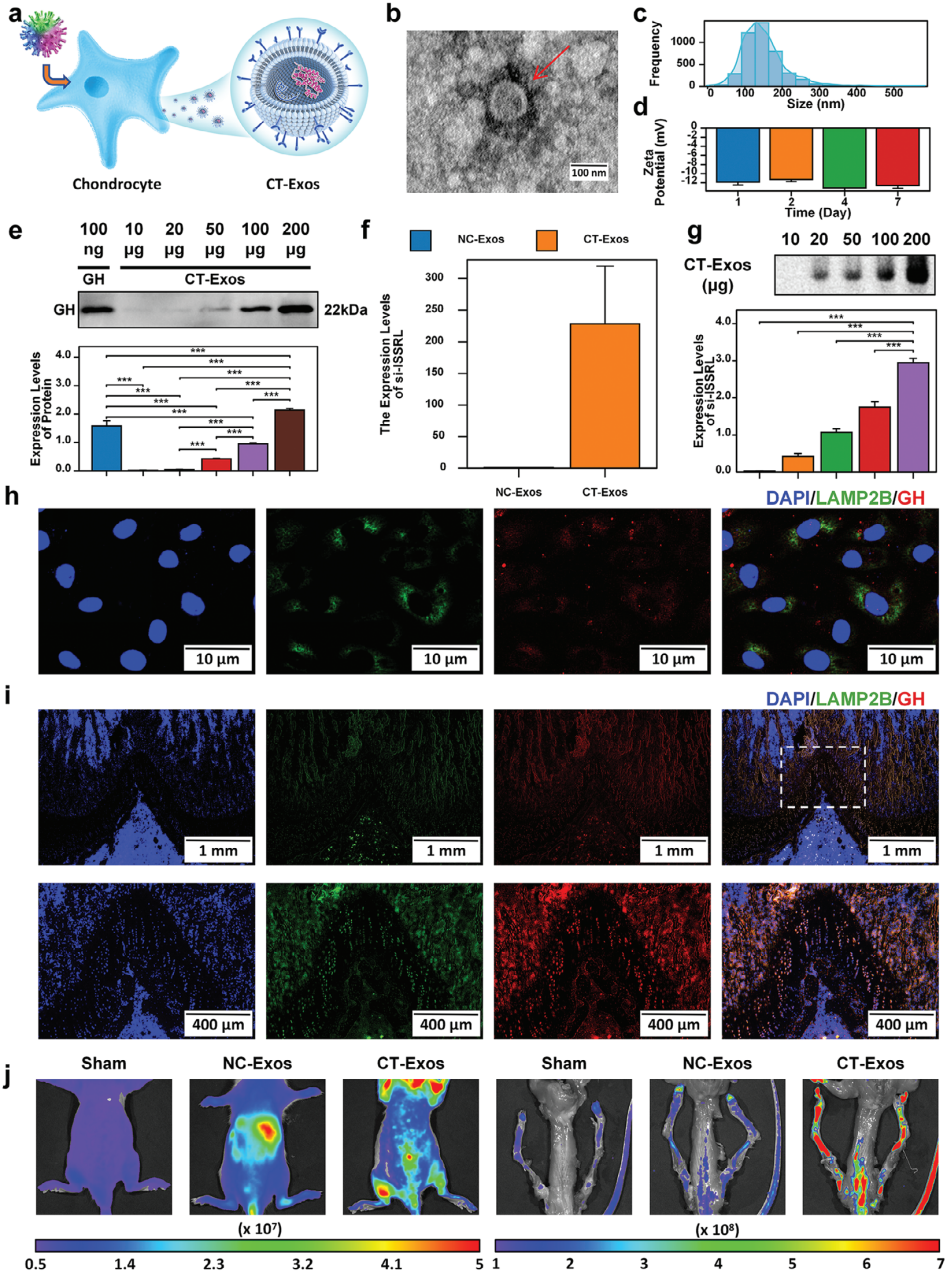
CT‐Exos targeted delivery of si‐ISSRL and GH to growth plate chondrocytes. a) A flowchart for obtaining CT‐Exos. b) The morphology of the extraction was examined via transmission electron microscope. c) Nano‐Sight analysis further verified that the size of extraction is approximately 100 nm. d) Zeta potential of CT‐Exos for 1, 2, 4, and 7 d. e) Western blotting analysis indicated that CT‐Exos were loaded with abundant GH. f) RT‐qPCR revealed that the expression levels of si‐ISSRL were significantly increased in CT‐Exos. g) Gel electrophoresis analyses the expression levels of si‐ISSRL in CT‐Exos. h) The CT‐Exos (WYRGRL/LAMP2b/EGFP, Green fluorescence; GH/mCherry, Red fluorescence) uptake by chondrocytes (Blue fluorescence) was confirmed. i) The CT‐Exos (WYRGRL/LAMP2b/EGFP, Green fluorescence; GH/mCherry, Red fluorescence) targeted delivery to the growth plate cartilage. j) The UCL intensity was noticeably enhanced in cartilage and liver in the NC‐Exos group, and the UCL significantly increased in cartilage while decreasing in the liver and other organs, in comparison to the NC‐Exos group. Two groups were compared using T‐test or three groups were compared using ANOVA followed by Tukey's test. *P < 0.05, **P < 0.01, ***P < 0.001 versus control.

Engineered exosomes (CT‐Exos) were obtained from chondrocytes following transfection with lentivirus (**Figure** **6**a). The morphology of the extracted exosomes was examined using transmission electron microscopy (Figure 6b). Nano‐Sight analysis further confirmed that the exosomes had a size of less than 100 nm (Figure 6c). The bands representing naked GH protein were detected in the CT‐Exos, and their expression levels were upregulated in accordance with exosome levels, and 150 µg CT‐Exos nearly loading 100 ng GH (Figure 6d). The expression of siRNA‐ISSRL in CT‐Exos was quantified using RT‐qPCR (Figure 6e). As demonstrated in Figure 6f, the band representing naked siRNA‐ISSRL. Subsequently, human chondrocytes were co‐cultured with CT‐Exos, and it was observed that exosomes and GH entered these chondrocytes (Figure 6g). Frozen tissue section results clearly show that CT‐Exos efficiently carries siRNA ISSRL and GH proteins for targeted delivery to the growth plate cartilage (Figure 6h).

To investigate the in vivo cartilage‐targeting ability of CT‐Exos, bioluminescence imaging was performed using an IVIS Lumina Series III (Perkin Elmer). The upconversion luminescence (UCL) and frozen tissue section fluorescence intensity were noticeably enhanced in cartilage and other organs, especially the liver in the NC‐Exos group (Figure 6i and Figure S4, Supporting Information). Following the injection of CT‐Exos into the rat tail vein, UCL increased significantly in cartilage while decreasing in the liver and other organs, compared to the NC‐Exos group (Figure 6i and Figure S4, Supporting Information). The absence of UCL and fluorescence in other tissues within the CT‐Exos group provided further evidence that the CT‐Exos modification endowed them with specific targeting capabilities toward the growth plate cartilage.

### 2.8 CT‐Exos Counteracting ISSRL's Inhibitory Effects on Chondrocyte Proliferation and Bone Formation In Vitro

**Figure 7:**
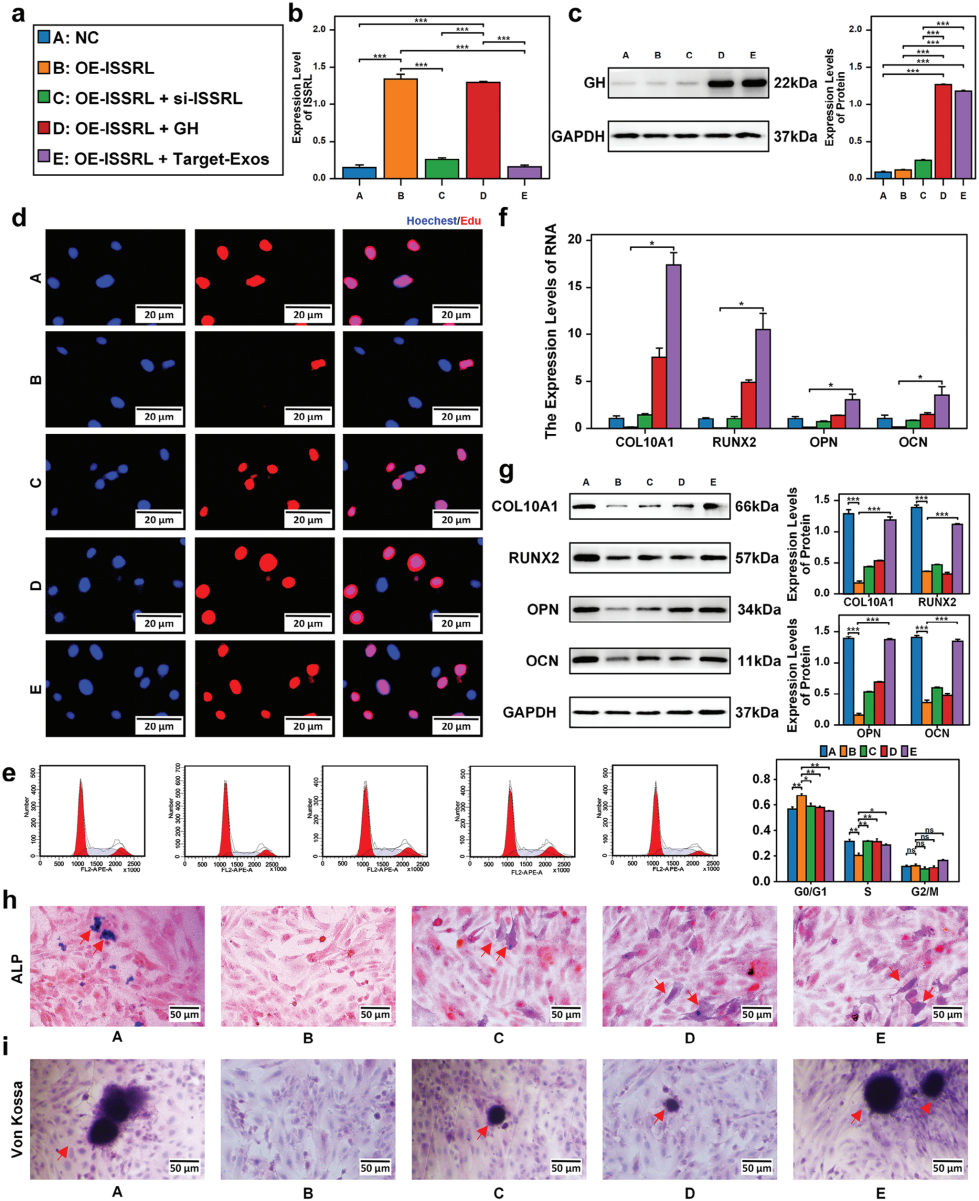
CT‐Exos counteracting inhibitory effects of ISSRL overexpression on chondrocyte proliferation and bone formation in vitro. a) Experimental grouping: Group A (Blue): Normal chondrocyte (NC). Group B (Orange): Chondrocyte with ISSRL overexpression (OE‐ISSRL). Group C (Green): ISSRL‐overexpressed chondrocyte with simultaneous ISSRL expression increase using si‐ISSRL. Group D (Red): ISSRL‐overexpressed chondrocyte with concurrent upregulation of GH using GH protein. Group E (Purple): ISSRL‐overexpressed chondrocyte co‐cultured with CT‐Exos. b) RT‐qPCR revealed that ISSRL expression was increased in group B and D, and rescued in group C and E. c) Western blot showed that GH protein expression was up‐regulated in group D and E. d) EdU showed that in group B, chondrocyte proliferation was diminished, but this trend was reversed in group C, D, and E. e) Flow cytometric analysis revealed that cell cycle was arrested in the G0/G1 phase in group B, more chondrocyte in the M2/S phase in group C, D, and E. f) The RT‐qPCR results revealed that mRNA expression levels of COL10A1, RUNX2, OPN, and OCN were reduced in group B, with no significant differences were observed between group C, D or E and group A. g) The western blot outcomes indicated that protein expression levels of COL10A1, RUNX2, OPN, and OCN were reduced in group B, with no significant differences were observed between group C, D or E and group A. h) ALP activity declined in group B but recovered in group C, D, and E. i) Von Kossa staining indicated reduced mineralization in group B, whereas mineralization was regained in group C, D, and E. The data are presented as the mean±SD. *n* = 3. Five groups were compared using ANOVA followed by Tukey's test. *P < 0.05, **P < 0.01, ***P < 0.001 versus control.

Experimental grouping (**Figure** **7**a): group A, there was NC chondrocyte (NC); group B, there was chondrocyte with ISSRL overexpression (OE‐ISSRL); group C, OE‐ISSRL chondrocyte was cocultured with siRNA‐ISSRL (GCTGACCGAGTCTGGAGAT, 100 × 10^−9^ m, Guangzhou RiboBio Co.); group D, OE‐ISSRL chondrocyte was cocultured with GH protein (100 ng mL^−1^, Proteintech, Cat No. HZ‐1007). Group E, purple color was ISSRL overexpressed chondrocyte, co‐cultured with CT‐Exos (150 µg mL^−1^).

The application of CT‐Exos led to the downregulation of ISSRL expression and simultaneous upregulation of GH expression in human chondrocytes following ISSRL overexpression via lentiviral vectors (*P*‐value < 0.05, Figure 7b,c). This intervention significantly mitigated the inhibitory impact of ISSRL overexpression on chondrocyte proliferation and bone formation (Figure 7d‐i).

As depicted in Figure 7d, the EdU assay indicated that si‐ISSRL, GH, and CT‐Exos promoted the proliferation of human chondrocytes. Flow cytometry analysis revealed that the number of G0/G1‐phase cells in the si‐ISSRL, GH, and CT‐Exos‐treated group was notably lower than in the OE‐ISSRL group (Figure 7e). Furthermore, a higher proportion of cells were in the S and G2/M phases in the si‐ISSRL, GH, and CT‐Exos‐treated group compared to the OE‐ISSRL group, underscoring the greater prominence of G1 cell cycle arrest in the si‐ISSRL, GH, and CT‐Exos‐treated cohort (Figure 7e).

To assess the influence of CT‐Exos on endochondral ossification and chondrocyte hypertrophy, the expressions of COL10A1, RUNX2, OCN, OPN, Von Kossa staining, and ALP were measured following CT‐Exos treatment. Western blot and RT‐qPCR analyses revealed significant upregulation of COL10A1 and RUNX2 expression levels compared to si‐ISSRL and GH (Figure 7f,g), indicating the promotion of chondrocyte hypertrophy. Following CT‐Exos treatment, the expression of osteogenic genes (OCN and OPN) was enhanced compared to si‐ISSRL and GH (Figure 7f,g). Additionally, ALP activity was increased (Figure 7h). Moreover, as demonstrated in Figure 7i, Von Kossa staining showcased heightened mineralization. Collectively, these findings underscore the significant promotion of hypertrophy, endochondral ossification, and chondrocyte proliferation by CT‐Exos in vitro.

### 2.9 CT‐Exos: Overcoming ISSRL‐Mediated Inhibition of Chondrocyte Proliferation and Bone Formation, Restoring Height in Rats In Vivo

**Figure 8:**
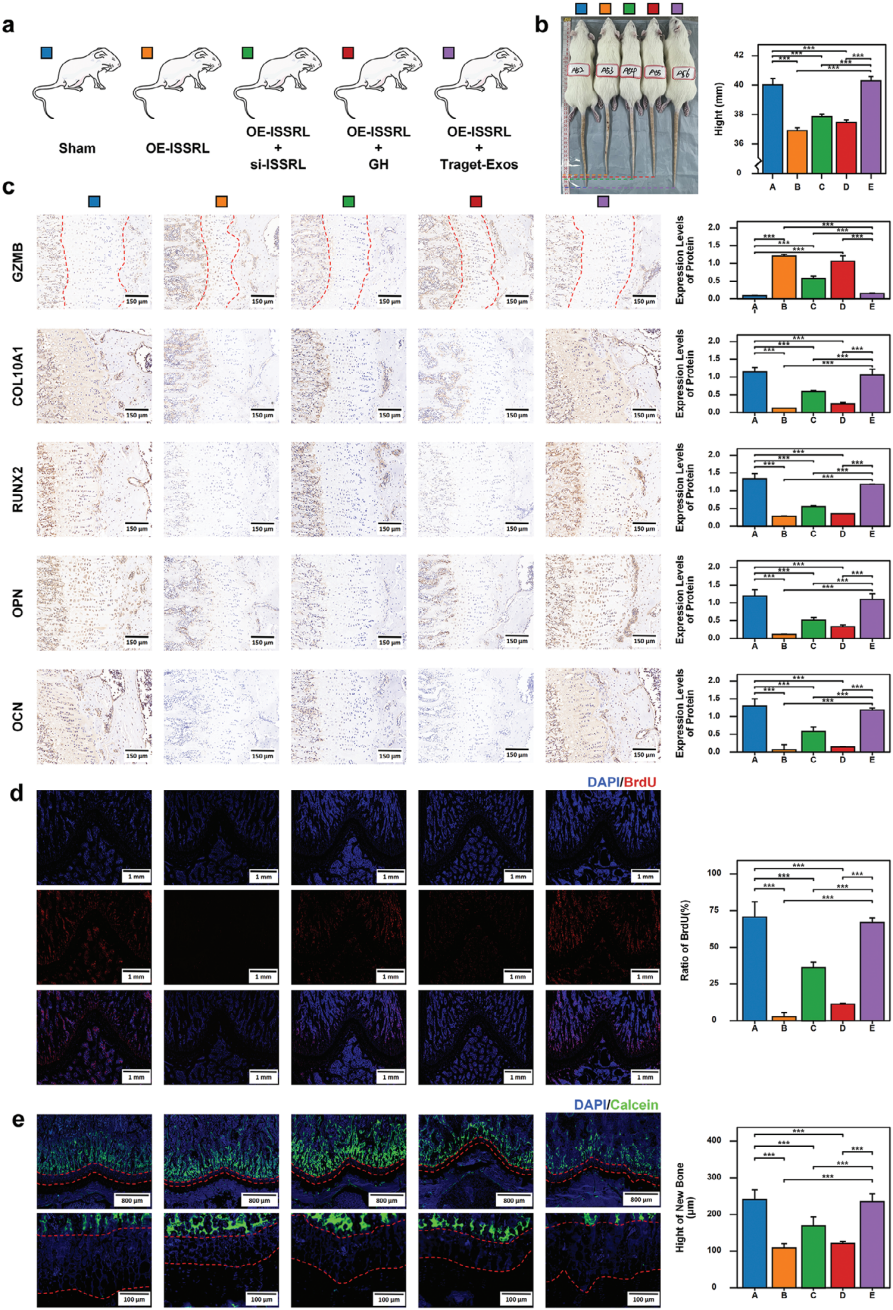
CT‐Exos: Overcoming ISSRL‐mediated inhibition of chondrocyte proliferation and bone formation, restoring height in rats In Vivo. a) Experimental grouping: Group A (Blue): Sham (NC). Group B (Orange): Rats with ISSRL overexpression (OE‐ISSRL). Group C (Green): ISSRL‐overexpressed rats with concurrent si‐ISSRL injection. Group D (Red): ISSRL‐overexpressed rats with concurrent GH protein injection. Group E (Purple): ISSRL‐overexpressed rats with CT‐Exos injection. b) Overexpression of ISSRL resulted in a short stature phenotype in rats, CT‐Exos significantly recovered the stature of rats. c) The expression of GZMB was up‐regulated after overexpression of ISSRL, but it was inhibited in group E. The expression of COL10A1, RUNX2, OPN, and OCN was decreased after overexpression of ISSRL, and the expression levels were increased in group E. d) BrdU labeling confirmed the inhibitory effect on proliferation within the growth plate cartilage of group B and improved in group E. e) Calcein staining confirmed the inhibitory effect on bone formation within the growth plate cartilage of group B and improved in group E. The data are presented as the mean±SD. *n* = 3. Five groups were compared using ANOVA followed by Tukey's test. *P < 0.05, **P < 0.01, ***P < 0.001 versus control.

Given the promising in vitro treatment potential of CT‐Exos, an in vivo study was conducted using an SD rat model (OE‐ISSRL) to investigate its therapeutic role in treating short stature. The rat model of short stature is created by introducing lentiviral vectors that overexpress ISSRL into immature wild‐type rats through tail vein injection (**Figures** **8**b and S5, Supporting Information). The short‐stature rats were divided into five groups: NC, OE‐ISSRL, OE‐ISSRL + si‐ISSRL (GCTGACCGAGTCTGGAGAT, 5 nmol once four days, Guangzhou RiboBio Co.), OE‐ISSRL + GH (100 ng per day), and OE‐ISSRL + CT‐Exos (450 µg, once 3 d) group (Figure 8a). A negative control (NC) group received an NC‐lentivirus vector via tail vein injection. Examination of the visceral organs through HE staining indicated the in vivo biocompatibility of OE‐ISSRL + CT‐Exos, particularly in the CT‐Exos group when compared to the NC group (Figure S6, Supporting Information).

RT‐qPCR unequivocally confirmed a substantial downregulation of miR‐877‐3p and a remarkable upregulation of ISSRL and GZMB in the growth plate cartilage of SD rats within the OE‐ISSRL group, when compared to the NC group (Figure S7, Supporting Information). Simultaneously, analyses conducted via IHC, RT‐qPCR, and western blot unveiled a significant suppression in the expression of key markers associated with chondrocyte hypertrophic differentiation, such as COL10A1 and RUNX2, along with osteogenic gene markers like OPN and OCN in the OE‐ISSRL group in comparison to the NC group (Figure 8c and Figure S8, Supporting Information). Moreover, BrdU labeling and Calcein staining definitively confirmed the inhibitory effect on proliferation and bone formation within the growth plate cartilage of the OE‐ISSRL group, as opposed to the NC group (Figure 8d,e). Consequently, the rats manifested a distinctive phenotype characterized by short stature.

We aimed to counteract the inhibition of proliferation and bone formation in the growth plate of rats induced by OE‐ISSRL by administering growth hormone. However, as evident from the experimental outcomes in the OE‐ISSRL + GH group, growth hormone proved ineffective in reversing the downregulation of COL10A1, RUNX2, OPN, and OCN expression induced by OE‐ISSRL (Figure 8c and Figure S8, Supporting Information). Furthermore, BrdU labeling and Calcein staining confirmed the lack of restoration in the proliferation and bone formation of the growth plate cartilage following GH treatment (Figure 8c and Figure S8, Supporting Information). Consequently, the rats continued to exhibit a stunted phenotype. These findings suggest that the aberrant expression of long non‐coding RNA remained unalleviated, leading to resistance to growth hormone therapy.

This is an exceptionally intriguing discovery. The authors have observed that, in contrast to the NC group, the OE‐ISSRL + CT‐Exos group showcased the restoration of COL10A1, RUNX2, OPN, and OCN expression, which had been downregulated by OE‐ISSRL (Figure 8c and Figure S8, Supporting Information). Additionally, both the proliferation and bone formation of the growth plate cartilage in the rats returned to normal (Figure 8d,e). What is particularly noteworthy is the exceptional efficacy of the OE‐ISSRL + CT‐Exos group in treating short stature, outperforming the results seen in the OE‐ISSRL + si‐ISSRL group (Figure 8 and Figures S5 and S8, Supporting Information). These findings strongly suggest that the targeted delivery of siRNA to the growth plate cartilage to alleviate the inhibitory effects of abnormal long non‐coding RNA, combined with the precision delivery of growth hormone, constitutes an effective therapeutic strategy for restoring normal stature.

## 3 Discussion

It is widely acknowledged that circulating molecules play a critical role in governing bone development within the growth plate cartilage. Given the abundance of exosomes in circulating plasma, comprehending the biological role of plasma exosomes is crucial for deciphering the molecular underpinnings of conditions like ISS effects. Our study aimed to evaluate the impact of plasma exosomes on ISS pathogenesis by coculturing normal human chondrocytes with plasma exosomes from ISS patients. Through a fluorescent tracer assay, it was confirmed that exosomes indeed entered human chondrocytes, leading to the intriguing discovery of inhibited chondrocyte proliferation and bone formation upon exposure to these exosomes.

Considering the well‐established association between lncRNAs and cartilage‐related processes such as development, degeneration, regeneration, and the pathogenesis of conditions like osteoarthritis,^[^ ^16^, ^17^, ^18^ ^]^ our investigation commenced with an analysis of the differential lncRNA expression profile within plasma exosomes of ISS patients and normal control children, and 99 DELs were obtained (53 upregulated and 46 downregulated). Further scrutiny focused on the top 40 log_2_(fold change) DELs, highlighting the pronounced significance of ISSRL. Importantly, ISSRL exhibited a specificity of 95.16%% and a sensitivity of 95.16% in distinguishing children with ISS from normal controls (Cut‐off value: 1.9266, AUC: 0.975, CI 95%: 0.984‐1.000). These findings underscore ISSRL as a credible biomarker for diagnosing ISS and potentially as a therapeutic target. Positioned as a lncRNA between the UQCRHP2 and RAB10 genes on chromosome 2p23.3, the biological functions of ISSRL remain unexplored in the existing literature.

To unravel the biological role of the upregulated ISSRL within plasma exosomes, we conducted experiments that involved the downregulation of ISSRL expression following the coculture of human chondrocytes with ISS patient‐derived exosomes. The ensuing rescue tests provided clear evidence that the attenuation of ISSRL significantly alleviated the inhibition of chondrocyte proliferation and bone formation induced by ISS exosomes. Subsequently, we enhanced the expression of ISSRL in human chondrocytes using the OE‐ISSRL plasmid, independent of exosomes. This manipulation once again resulted in suppressed chondrocyte proliferation and bone formation, indicating that the heightened levels of ISSRL within plasma exosomes are responsible for the observed inhibition of these processes.

In situ hybridization demonstrated the cytoplasmic localization of ISSRL in chondrocytes, suggesting that its biological function might be exerted via the mechanism of miRNA sponging.^[^ ^19^, ^20^, ^21^ ^]^ Subsequent mRNA microarray analysis, ceRNA network analysis, RT‐qPCR, and western blotting identified potential candidate miRNA (miR‐877‐3p) and mRNA (GZMB) for further investigation. Prior research has explored the regulatory roles of miR‐877‐3p in various contexts; however, its impact on chondrocytes remains unexplored.^[^ ^22^, ^23^, ^24^ ^]^ Our study initially confirmed the co‐localization of ISSRL and miR‐877‐3p in rat femoral growth plates and human chondrocytes using Fluorescence In Situ Hybridization. Furthermore, molecular docking predictions, RIP assays, and luciferase reporter assays indicated that miR‐877‐3p could potentially target ISSRL through AGO2. Subsequent rescue experiments demonstrated that miR‐877‐3p overexpression countered the suppression of chondrocyte proliferation and bone formation induced by ISSRL upregulation. Additionally, previous studies have suggested GZMB's involvement in human articular chondrocytes and its potential role in cartilage proteoglycan degradation.^[^ ^25^, ^26^ ^]^ Our investigation aligned with these findings, as GZMB upregulation resulted in the suppression of aggrecan and COL2A1 expression in human chondrocytes, ultimately contributing to cartilage proteoglycan degradation. Consequently, chondrocyte proliferation and bone formation were hindered. Rescue experiments further validated that the inhibitory effect of ISSRL on chondrocyte proliferation and bone formation occurred through the miR‐877‐3p/GZMB axis.

Exosomes, a subset of extracellular vesicles, have proven to be effective therapeutic agents for various diseases.^[^ ^27^ ^]^ Engineered exosomes refer to exosomes that have been modified both at their surface and internally with therapeutic molecules.^[^ ^28^ ^]^ Following appropriate modifications, engineered exosomes demonstrate the capability to precisely deliver drugs to the targeted lesion site, thereby holding substantial promise for future applications.^[^ ^29^ ^]^ A previous study introduced a cardiac homing peptide (HHP) onto the surface of extracellular vesicles derived from cardiac myocytes by engineering the fusion of HHP with the N‐terminus of lysosomal‐associated membrane protein 2b (LAMP2b), achieving cardiac‐specific delivery of extracellular vesicles.^[^ ^30^ ^]^ Intriguingly, articular cartilage primarily consists of the dense COL2A1, and it was reported that WYRGRL (Trp‐Tyr‐Arg‐Gly‐Arg‐Leu), one of the collagen II‐targeting peptides, resulted in a 72‐fold increase in cartilage‐targeting efficiency for the peptide‐functionalized nanoplatform in vivo.^[^ ^31^, ^32^ ^]^

In the current research, we selected to fuse the WYRGRL peptide to the N‐terminus of LAMP2b to enable targeted delivery to the growth plate cartilage of siRNA‐ISSRL and GH. CT‐Exos were isolated from chondrocytes transfected with lentivirus, loaded with si‐ISSRL and GH, and exhibiting abundant LAMP2b‐WYRGRL cartilage‐targeting peptides on their surface. Subsequently, human chondrocytes were cocultured with CT‐Exos carrying si‐ISSRL and GH‐Mcheery. The results demonstrate that CT‐Exos effectively enters chondrocytes, reversing the inhibitory effects of ISSRL on the chondrocyte's proliferation and bone formation. In vivo bioluminescence imaging revealed a significant enhancement in cartilage‐targeting with CT‐Exos compared to NC‐Exos. This observation was further validated through in situ hybridization within the rat's growth plate cartilage.

To assess the efficacy of CT‐Exos carrying si‐ISSRL and GH in treating short stature, in vivo experiments were conducted. Initially, immature rats were subjected to overexpression of ISSRL through tail vein injection of lentiviral vectors, resulting in a short stature phenotype. Subsequently, we employed si‐ISSRL lentiviral vectors, growth hormone (GH), and CT‐Exos carrying si‐ISSRL and GH to treat the rat with short stature. An intriguing finding emerged as we observed that administering GH alone failed to ameliorate the short stature induced by ISSRL overexpression. These results suggest that ISSRL overexpression might lead to GH resistance, potentially being a critical clue to the poor efficacy of GH therapy in children with ISS. In contrast to our expectations, in vivo administration of si‐ISSRL to counteract ISSRL only demonstrated a modest effect in alleviating chondrocyte proliferation, bone formation, and the short stature caused by ISSRL overexpression. We speculate that this outcome could be attributed to factors such as siRNA degradation by nucleases in the circulatory system and a lack of targeting, resulting in insufficient delivery to the growth plate.

A highly significant discovery was made, where we observed that CT‐Exos specifically targets the growth plate cartilage, delivering si‐ISSRL to suppress ISSRL expression and increasing the local GH concentration in the growth plate cartilage. This led to a marked enhancement in chondrocyte proliferation, bone formation, and the resolution of short stature resulting from ISSRL overexpression. The current study introduces a novel therapeutic strategy for addressing ISS, wherein extracellular vesicle targeting technology is employed to deliver siRNA, thereby mitigating the inhibitory effect of abnormally expressed non‐coding RNA on growth plate cartilage development, while precisely delivering GH to promote bone growth and correct the short stature phenotype.

The ISS definition suggests that it encompasses a group of patients with unexplained short stature. The primary treatment for ISS currently involves the administration of recombinant growth hormone (rhGH). However, unlike growth hormone deficiency‐related short stature, individuals with idiopathic short stature do not lack growth hormone. When rhGH is administered to children with ISS, it affects not only the cartilage but also various organs involved in the GH‐IGF‐I axis. As a result, the long‐term use of rhGH in ISS children has raised concerns among researchers. For instance, Carel et al.^[^ ^10^ ^]^ and Ying et al.^[^ ^11^ ^]^ reported an elevated mortality rate in ISS patients treated with rhGH, attributing it to complications such as subarachnoid or intracerebral hemorrhage, cardiovascular diseases, hyperglycemia, hyperinsulinemia, and bone cancers. Additionally, the effectiveness of rhGH treatment varies significantly across studies, offering limited benefits while imposing a substantial economic burden. Silvers et al.^[^ ^12^ ^]^ highlighted the increasing use of rhGH, estimating over 500000 potential U.S. children with ISS and a corresponding market potential exceeding $10 billion per year. This places a significant economic burden on the families of ISS children.

The current ISS etiology remains elusive, and resistance to treatment is a common challenge encountered during rhGH therapy. The present study discerned that the overexpression of ISSRL in rats resulted in resistance to growth hormone therapy, hindering the restoration of normal height. Conversely, employing small interfering RNA (siRNA) to inhibit ISSRL overexpression before initiating growth hormone therapy enabled the rats to achieve normal height recovery. This suggests that elevated ISSRL levels in ISS patients may constitute a clinical factor contributing to resistance to rhGH therapy, although further research is imperative for confirmation.

Various carriers for in vivo siRNA delivery exist, including adenovirus vectors, liposomes, and exosomes.^[^ ^33^ ^]^ In contrast to viral vectors, exosomes do not integrate into the patient's chromosomes, exhibit no immunogenicity, offer transient gene overexpression, and ensure high safety.^[^ ^34^ ^]^ Despite the straightforward manufacturing process and scalability of liposomes, they are associated with heightened toxicity and inflammation compared to exosomes. Hence, the authors selected exosomes as carriers, implementing targeted modifications to facilitate the delivery of siRNA and growth hormone to the growth plate. This strategic approach aims to counteract the inhibitory effects of abnormally expressed ISSRL on growth plate development, concurrently targeting the delivery of growth hormone. Theoretically, this minimizes the systemic complications associated with general growth hormone administration. The presented advancement holds promise for enhancing the future prospects of ISS treatment.

However, the current study also exhibits certain limitations. For instance, according to the LNCipedia database, lnc‐RAB10‐2:1 (ISSRL) is exclusively expressed in Homo sapiens and has not been detected in Chimpanzees, Mice, Drosophila, or zebrafish as of yet. Nevertheless, the NONCODE database reveals the presence of conserved sequences of ISSRL in species such as Rats, Mice, Rhesus, etc., in addition to its discovery in the human brain, heart, and testes. Considering the discrepancy in ISSRL expression conservation between the two databases, we conducted ISSRL FISH staining on frozen tissue sections of rat growth plate cartilage. The fluorescence signals detected confirmed the expression of ISSRL in the growth plate cartilage area, as illustrated in Figure 4m. Notably, following the injection of the OE‐ISSRL lentivirus into the tail vein of SD rats, there was an inhibition of proliferation and ossification in the growth plate cartilage, leading to the manifestation of the ISS phenotype. RT‐qPCR analysis of the growth plate tissue further validated the lentivirus‐induced upregulation of ISSRL, specifically in the cartilage region of ISS SD rats (Figure S7b, Supporting Information). Additionally, our experimental findings suggest that ISSRL within plasma exosomes can inhibit proliferation and ossification in human and rat chondrocytes via miR‐877‐3p/GZMB. However, the origin of these exosomes in ISS patients remains ambiguous, necessitating further comprehensive investigation in subsequent studies.

## 4 Conclusion

In conclusion, our investigation affirms that ISSRL demonstrates high specificity and sensitivity in distinguishing children with idiopathic short stature (ISS) from normal control children. Notably, it is the first study to establish that plasma exosomes from ISS‐affected children can impede the proliferation and bone formation of chondrocytes and growth plates through the action of lncRNAs. Both in vitro and in vivo findings illustrate that the elevated ISSRL, originating from plasma exosomes, can interact with miR‐877‐3p, regulate the GZMB axis, and consequently reduce chondrocyte and growth plate proliferation as well as bone formation. As a result, the distinctive trait of short stature is established. Subsequently, we engineered CT‐Exo‐siISSRL‐oeGH, successfully achieving targeted delivery of siRNA and GH to the growth plate cartilage, effectively mitigating the short stature phenomenon. Our research sheds new light on the diagnosis and treatment of ISS. Identifying the role of plasma exosomes in ISS development may provide a unique therapeutic avenue for ISS and should be further explored in future studies through multicenter collaboration.

## 5 Experimental Section

### Study Subject Characteristics and Plasma Exosomes Extraction

Sixty‐two patient pairs suffering from ISS and age/gender‐matched controls were involved from Nanchang University's Second Affiliated Hospital in China. The 62 ISS children, including 28 males and 34 females, median (IQR) of age was 9.75 (7.75, 12) and height was 128.95 (113.25138.85). There were 32 males and 30 females in the matched control group, with a median (IQR) age of 9.65 (8.4, 10.5) and a median (IQR) height of 137.1 (131, 141.65) (**Table** **1**). Individuals with the mentioned conditions were not allowed to participate (i) skeletal anomalies or dysmorphic features in their neonatal duration, (ii) hormonal abnormalities (e.g., GH deficiency at a peak level of 6 ng mL^−1^) or thyroid or pubertal disorders, (iii) exposure to environmental factors and chronic elements that impact growth in humans, (iv) chromosomal aberrations that have been cytogenetically detected and (vi) whole exon sequencing.

**Table 1:** The baseline data table of patients.

Characteristics	ISS	NC	*P* value	Method
N	62	62		
Gender, *n* (%)			0.472	Chi sq test
Female	34 (27.4%)	30 (24.2%)		
Male	28 (22.6%)	32 (25.8%)		
Age, median (IQR)	9.75 (7.75, 12)	9.65 (8.4, 10.5)	0.932	Wilcoxon
High, median (IQR)	128.95 (113.25, 138.85)	137.1 (131, 141.65)	< 0.001	Wilcoxon
Weight, median (IQR)	26.15 (18.05, 33.4)	33.55 (29.15, 38.9)	< 0.001	Wilcoxon

Between October 2016 and March 2019, 62 pairs of blood samples were collected from participants under investigation. Whole venous blood samples were collected in EDTA tubes. The whole blood samples were centrifuged at 1900 *g* for 10 min at 4 °C with no brake. The supernatant samples were then centrifuged 3000 *g* for 15 min at 4 °C. The resulting supernatant samples were then used for isolating exosomes. According to the manufacturer's instructions, the plasma isolated above was used to isolate exosomes using the Exosome Isolation and Purification Kit (from plasma or serum) plus (Umibio, Cat No. UR52151). The samples were then stored at −80 °C for long‐duration storage. LncRNAs microarray analysis was performed on three specimen pairs, whereas all 124 samples were employed to confirm lncRNA expression using quantitative reverse transcriptase‐polymerase chain reaction (RT‐qPCR). The Human Research Ethics Committee of Nanchang University's Second Affiliated Hospital gave its approval to the gathering of human specimens (No. Review [2016] No, (090)). Because a part of the participants were under the age of 14, their parents or legal guardians had to give written informed consent before they could participate.

### Microarray Analysis of Differentially Expressed lncRNAs

Extraction and purification of total RNA were carried out using the exoRNeasy Serum/Plasma Midi Kit (QIAGEN, GmBH, Germany) in the light of the instructions detailed by the manufacturer, and an Agilent Bioanalyzer 2100 was used to check for a RIN number for inspecting RNA integration (Agilent technologies, Santa Clara, CA, USA). Following the manufacturer's instructions, amplification of total RNA was carried out which was later labeled using the Low Input Quick Amp Labeling Kit, One‐Color (Agilent Technologies, Santa Clara, CA, US). The RNeasy small kit was used to purify labeled cRNA (QIAGEN, GmBH, Germany). Making use of the manufacturer‐provided instructions, individual slides were hybridized with 1.65 g Cy3‐labeled cRNA using the Gene Expression Hybridization Kit (Agilent Technologies, Santa Clara, CA, USA) in the Hybridization Oven (Agilent technologies, Santa Clara, CA, US). The Agilent Microarray Scanner (Agilent Technologies, Santa Clara, CA, USA) was employed for scanning the slides with the default settings: Dye channel: Green, Scan resolution is 3m, PMT is 100%, and the bit depth is 20 bits. Feature Extraction software 12.0 was used to extract the data (Agilent Technologies, Santa Clara, CA, USA). Quantile method, limma packages in R, was used to standardize the raw data. Three sets of microarray assays were performed for the control group and three sets for the ISS group. Identification of the differentially expressed lncRNAs (DELs) was made using Fold Change filtering and the Student's t‐test (log_2_(fold change) ≥ 2.0, *P*‐value < 0.05).

The target genes of DELs were identified using Cis and Trans regulated predicted.^[^ ^35^ ^]^ The cellular components (CC), and molecular functions (MF), and biological processes (BP) of the identified target genes of DELs were assessed using gene ontology (GO) enrichment analysis (http://www.geneongoloty.org/). Meanwhile, to discover molecular pathways, DAVID (6.8; https://david.ncifcrf.gov/) was selected for a KEGG analysis. In KEGG and GO analysis, the enrichment scores of ‐log10 (*P*‐value) were utilized as the cutoff value.

### mRNA High‐Throughput Sequencing and Bioinformatics Analyses

After the extraction of total RNA using the Eastep Super RNA easy Total RNA Kit (Promega (Beijing) Biotech Co., Ltd.), the TruSeq Stranded mRNA LT Specimen Prep Kit (Illumina, San Diego, CA, USA) was used to build the libraries as stated by the instructions obtained from the manufacturer. The transcriptome sequencing and analysis were subsequently conducted using OE Biotech Co., Ltd. (Shanghai, China). The Illumina HiSeq X Ten sequencer was used to complete the sequence. Trimmomatic was used to obtain high‐quality clean reads after adapter‐containing, ploy‐N‐containing, and low‐quality reads were deleted. HISAT2 was used to compare clean readings to the human genome. The FPKM value of each gene was quantified by Cufflink. The read counts of each gene were calculated using HTSeq‐count. The differential expression was discovered using the R program DESeq (2012). For GO and KEGG analyses, a *P*‐value less than 0.05 and a log_2_(fold change) larger than 3 were used as cutoff values.

Longitudinal bone development is regulated by miRNAs, and lncRNAs can serve as miRNA sponges by changing levels of gene expression. As a result, the previous methods were used to construct and visualize the lncRNA‐miRNA‐mRNA (ceRNA) regulatory network.^[^ ^35^ ^]^

### In Situ Hybridization

Incubation of the human chondrocytes was completed for 48 h at 65 °C with 500 ng mL^−1^ of FAM‐labeled probe and Cy3‐labeled probe (ISSRL 5′‐FAM‐TTTACACATTTCTCCAACTGCTGGCATTGCTCTCTCACTGTTGTTA‐3′, length 46 bp; hsa‐miR‐877‐3p 5′‐Cy3‐ CTGGGAGGAGGGAGAAGAGGA‐3′, length 21 bp) in the light of instructions outlined by the manufacturer to examine the distribution of ISSRL and hsa‐miR‐877‐3p expression (miRCURY LNA miRNA ISH kit, Thermo Fisher, Shanghai, China).

Six 6 week old rats with a weight ranging from 16.4‐22.5 g were used. Femur samples were taken from the rats after euthanization. Following that, the femur samples were decalcified for 4 weeks with a 10% EDTA solution. Then, one specimen was cut into two equal coronal sections. The femur specimens were embedded within paraffin. 4 m thick sliced pieces of the paraffin‐embedded specimens were then sliced following which in situ hybridization was subsequently carried out (miRCURY LNA miRNA ISH Kit; Thermo Fisher Scientific, Inc.).

### Cell Culture, Exosomes Isolating

Procell life science technology co., Ltd (Wuhan, China) provided the human chondrocytes, which were cultivated in DMEM (Dulbecco's modified Eagle's medium; Gibco, Thermo Fisher Scientific, Inc.) with 10% FBS (Gibco, Australia) in a 37 °C humidified incubator under 5% CO_2_ atmosphere. Lentiviral vectors carrying si‐ISSRL‐WYRGRL/LAMP2b/EGFP‐GH/mCherry and si‐ISSRL‐WYRGRL/LAMP2b‐GH were constructed by VectorBuilder (Guangzhou, China). Transfection was performed when chondrocyte fusion reached approximately 30% to 40% of confluence. Stable transfected cells were selected using puromycin. Chondrocytes were cultured in media containing exosome‐depleted FBS, and the cell supernatant was collected after 24 h of cultivation. According to the protocol, NC‐Exos and cartilage target exosomes containing siRNA‐ISSRL and OE‐GH (CT‐Exos, CT‐Exo‐siISSRL‐oeGH) were isolated and purified using an Exosome Isolation Kit (from cell culture media) (Umibio, Cat No. UR52121). The BCA Protein Assay Kit (Beyotime, Cat No. P0010) was used for protein quantification.

### Cell Proliferation Assay

For the evaluation of cell proliferation, chondrocytes carrying the ISSRL were cultivated in a six‐well plate until reaching an 80% confluence. Subsequently, the chondrocytes were exposed to 5‐ethynyl‐2′‐deoxyuridine (EdU; sourced from Guangzhou Ribobio Company) for a duration of 2 h, meticulously adhering to the manufacturer's recommended protocol. Following this incubation period, the chondrocytes were subjected to a 1xApollo reaction mixture, amounting to 200 µL, for a duration of 25 min. In the subsequent step, Hoechst 33342 (utilized at a concentration of 5 µg mL^−1^) was employed for staining the chondrocytes, allowing for a staining duration of 30 min. Ultimately, the stained chondrocytes were meticulously scrutinized under a fluorescence microscope and subsequently captured via imaging.

### Overexpression Vectors ISSRL, miR‐877‐3p Mimic, and siRNA of ISSRL and GZMB

The ISSRL plasmid (GV367‐Ubi‐MCS‐SV40‐EGFP‐IRES‐puromycin) was constructed successfully by GENEchem Co., Ltd. (Figure S9, Supporting Information). A six‐well plate was used to culture the chondrocytes until 80% confluence. Using Lipofectamine 3000 (Invitrogen, Carlsbad, CA, USA). The ISSRL plasmid was transfected to a six‐well plate. An empty vector was considered as the control group. A fluorescence microscope was used to evaluate the transfection efficiency of the ISSRL. ISSRL existed as 50 × 10^−9^ m final concentration.

ISSRL siRNA (GCTGACCGAGTCTGGAGAT) and NC siRNA (AAGTCGGGTCAAGAGAAGC) were purchased from Guangzhou RiboBio Co., Ltd. Granzyme B (GZMB) siRNA (GCACCAAAGTCTCAAGCTT) and NC siRNA (AAGTCGGGTCAAGAGAAGC) were also purchased from Guangzhou RiboBio Co., Ltd. The miR‐877‐3p mimics (sense UGUCCUCUUC UCCCUCCUCCCA, and antisense UGGGAGGAGGGAGAAGAGGACA) and NC mimics (sense UUUGUACUACACAAAAGUACUG and antisense CAGUACUUUUGUGUAGU ACAAA) were obtained from Guangzhou RiboBio Co., Ltd. (China). Using Lipofectamine 2000 (Invitrogen, Carlsbad, USA). The miR‐877‐3p mimics (5 µL)/NC were transfected to a six‐well plate based on the instructions given by the manufacturer. miR‐877‐3p /NC mimics existed as a final concentration of 50 × 10^−9^ m. ISSRL siRNA (5 µL)/NC and GZMB siRNA (5 µL)/NC were transfected using Lipofectamine 2000 (Invitrogen, Carlsbad, USA) to a 6‐well plate, based on the protocol defined by the manufacturer. The final concentration of ISSRL siRNA(5 µL)/NC and GZMB siRNA/NC was 50 × 10^−9^ m. 72 h following the transfection of cells, further experimentation was carried out. The entire set of experiments was conducted thrice independently.

### RT‐qPCR and Western Blot Analysis

Extraction of the total RNA was made from each frozen specimen making use of an Eastep Super RNA Extraction Kit (Promega (Beijing) Biotech Co., Ltd.). The PrimeScript RT Reagent Kit with gDNA Eraser was then utilized to reverse the transcription (TaKaRa, Japan). The mRNA levels of OCN, OPN, COL10A1, COL2A1, RUNX2, GZMB, GABBR2, and proteoglycan were measured using the primers listed in Table S1 (Supporting Information) using an ABI Q6 PCR machine (Applied Biosystems, TFS Inc.). The following was the RT‐qPCR cycle (TB Green Premix Ex Taq II, TaKaRa, Japan): 10 minutes at 95 °C, followed by 40 cycles of 10 seconds at 95 °C and 34 s at 60 °C. As an internal control, GAPDH was used. According^[^ ^36^ ^]^ to the previous experimental method, RT‐qPCR was used to detect the expression level of miRNA in cell and animal tissue samples. The internal reference used was U6, and the corresponding primer sequence was already present in the Table S1 (Supporting Information). Each reaction was carried out in triplicate using three different cell preparations. The relative mRNA and miRNA expression of the individual gene after normalization was estimated using the 2‐Ct technique. The internal control was employed to eliminate possible errors caused by variations in RNA amount and transcription efficiency.

The protein specimens were extracted through cell lysis after the chondrocytes from the two groups were harvested (Tissue cell total protein extraction kit, Applygen Technologies Inc.). The BCA test was used to determine the total protein content (Thermo Fisher Scientific, Inc.). Using SDS‐PAGE (Beijing Biosynthesis Biotechnology, Beijing, China), the protein lysates were then separated. Then, the PVDF membrane was blocked using skim milk (Solarbio Inc.) for 1 h at room temperature and subsequently subjected to incubation with anti‐GZMB (1:1000 dilution; CST, USA), anti‐RUNX2 (1:1000 dilution; Abcam, USA), anti‐COL10A1 (1:1000 dilution; Abcam, USA), anti‐COL2A1 (1:2000 dilution; Abcam, USA), anti‐proteoglycan (1:2000 dilution; Abcam, USA), anti‐OPN (1:1000 dilution; Abcam, USA), and anti‐OCN (1:3000 dilution; Abcam, USA) primary antibodies. Following overnight incubation at 4 °C, 1 × TBST (Solarbio Inc.) was used and eventually set to incubate at room temperature for 1 h with HRP‐labeled rabbit anti‐mouse (1:3000 dilutions; Abcam, USA) secondary antibody. GAPDH (1:1000 dilution, Abcam, USA) and β‐actin (1:1000 dilution, Abcam, USA) was employed as an internal control. Using the WB Stripping buffer (NO:21059; Thermo Fisher SCIENTIFIC, USA) the membrane was reprobed with a new primary antibody if more than one protein was shown on the same Western blot membrane. Image Lab (v 5.2.1) measured the grey values.

### Von Kossa Staining and Alkaline Phosphatase Staining

Von Kossa staining Kit (Shanghai Gefan Biotechnology, Co., Ltd) was used to analyze the mineralized nodules from chondrocytes in vitro following the manufacturer's protocol. The Von Kossa and alkaline phosphatase staining procedures are discussed in the earlier report.^[^ ^37^ ^]^ Positive calcium nodules were recognized as black brown or dark black.

The chondrocytes were first washed thrice in PBS. The chondrocytes were then fixed for 15 min at room temperature with 4% paraformaldehyde. The fixed chondrocyte cells were then incubated in the dark for 48 h with a BCIP/NBT Alkaline Phosphatase Color Development Kit (Solarbio Inc.). The Alkaline Phosphatase Assay Kit was utilized for the detection of the chondrocyte cell lysates following the protocol defined by the manufacturer.

### Molecular Interaction Experiment—Luciferase Reporter Assay

Following the instructions provided by the manufacturer, the ISSRL plasmid, or its mutant portions, and the miR‐877‐3p mimic were cotransfected into 293T cells utilizing Lipofiter (Hanbio Biotechnology, Shanghai, China). It was decided that the structure based on the graphical presentation vector would be given preference. Similarly, cotransfection of GZMB plasmid (pSI‐Check2, Hanbio Biotechnology, Shanghai, China) and miR‐877‐3p was made into 293T cells, as were its mutant portions. The dual‐luciferase assay system (Promega Corporation) was chosen for assessing the transfected cells in terms of their firefly and Renilla luciferase activity, 48 h after transfection.

### Molecular Interaction Experiment—RNA Immunoprecipitation Assay

According to the manufacturer's instructions, RNA immunoprecipitation (RIP) was performed using the RNA binding protein immunoprecipitation kit (Millipore, 17‐701). In brief, chondrocytes were harvested and lysed in a complete RIPA buffer containing a mixture of protease and RNase inhibitors. Subsequently, the cell lysate was incubated with a human anti‐AGO2 antibody (Protentech, catalog number 67934‐1‐Ig) or a control normal IgG of the kit. The sample was then subjected to protease K digestion to isolate RNA from the immunoprecipitation. Finally, purified RNA was subjected to RT‐qPCR to confirm the presence of binding targets.

### In Vitro and In Vivo Bone Targeting

For in vivo growth plates targeting assessment, tail vein injections of CT‐Exo‐siISSRL‐oeGH, normal exosomes, and PBS were administered to 4 week old SD rats. At various time intervals, the animals were humanely euthanized, and imaging was performed on bones as well as organs including the heart, liver, spleen, lung, kidney, and intestines (excitation wavelength: 551 nm, emission wavelength: 567 nm).

### Animal Models—Animal Feeding and OE‐ISSRL Rats

The rats were fed a mixed diet (Beijing Keao Xieli Feed Company) at 22–24 °C under a 12:12 h light/dark cycle. The ISSRL plasmid (GV367‐Ubi‐MCS‐SV40‐EGFP‐IRES‐puromycin) was constructed successfully by GENEchem Co., Ltd. In group A, the 200 µL PBS was injected into the tail vein. ISSRL was overexpressed in 2 week rats via tail‐vein injection in groups B to E. In group C, 5 nmol siRNA of ISSRL was injected into the tail vein once every four days. In group D, intraperitoneal injection of GH into all SD rats was 100 ng every day. In group E, the CT‐Exos (300 µg) was injected into the tail vein once every 3 days. After the 30 cubs were fed to 6 week old, euthanization of the rats was carried out with administering excessive carbon dioxide. One‐third of the distal femur was resected and kept in 4% formaldehyde solution or liquid nitrogen for further use in immunehistochemistry, in situ hybridization, BrdU staining, calcein staining, and Western blot analysis. The flow chart (Figure S10, Supporting Information) demonstrates the detailed grouping information. The Animal Ethics Committee of Nanchang University (Nanchang, China) gave the formal ethical approval for animal experiments (No. NCULAE‐20221031060).

### Animal Models—Immunohistochemistry Analysis, BrdU and Calcein Staining

Femur and tibia samples of 6 week old rats were extracted after euthanasia. One‐third of the distal femur was resected and kept in 4% formaldehyde solution. The specimens were decalcified with 10% EDTA for 2 months. Then, one specimen was cut into two equal coronal sections. Subsequently, paraffin was employed to embed the coronal sections. 4 µm thickness, serial sections were obtained for safranin O, immunohistochemistry (IHC) analysis, BrdU and calcein staining. The flow chart (Figure S10, Supporting Information) demonstrates the detailed grouping information.

The proliferation rate of the femur growth plate was calculated via BrdU staining. BrdU (Sigma‐Aldrich, St. Louis, MO) was injected by intraperitoneal administration (150 ng g^‐1^) 2 h before the rats were euthanized. BrdU staining was performed using a BrdU In‐Situ Detection Kit (BD Biosciences, CA, USA), following by methyl green counterstaining. BrdU‐positive cells in the femur growth plate were counted and compared between the OE‐ISSRL group and the normal control group. The bone growth rate was counted by calcein staining. The calcein (Sigma‐Aldrich, MO, USA) was injected by intraperitoneal administration (150 ng g^‐1^) 7 d before the rats were euthanized. After rehydrating and counterstaining with DAPI, the sections of the distal femur were mounted with Fluoromount G (TFS Inc.). After fluorescence microscopic analysis (Keyence BZ‐X700; Keyence Corp., Osaka, Japan), the longitudinal growth of bone was calculated by measuring the distance between the respective green fluorescent bands (casein label) and the metaphyseal chondro‐osseous junction.

### Statistical Analysis

The statistical analysis methods for clinical data are summarized in Table 1. In in vitro and in vivo experiments, group comparisons were performed using the T‐test for two groups and ANOVA followed by Tukey's test for three or more groups. The correlation was assessed via Pearson correlation analysis. To further analyze the diagnostic efficiency of ISSRL in discriminating ISS group from normal control group, a receiver operating characteristic (ROC) analysis was conducted. The ROC curve and the area under the ROC curve (AUC) were calculated using an online tool, Xiantao Academic (https://www.xiantaozi.com/products). *P*‐values of less than 0.001 (***), 0.01 (**) and 0.05 (*) were deemed as significant statistically. Not significant was ns. All statistical tests were carried out using Xiantao Academic.

### Ethics Statement

Ethical approval for this research was obtained from the Human and Animal Research Ethics Committee of the Second Affiliated Hospital of Nanchang University (Nanchang, China) (No. Review [2016] No, (090)). The Animal Ethics Committee of Nanchang University (Nanchang, China) gave the formal ethical approval for animal experiments (No. NCULAE‐20221031060).

## Conflict of Interest

The authors declare no conflict of interest.

## Author Contributions

J.J.Y. and W.X.H. designed the study; J.J.Y., L.X.J., Y.J.H., W.Y.M., L.S.Q., Z.J.Q., Z.X.W., H.G.W., H.Y.Z, C.X.G., and Z.W.R. conducted the experiment; W.Z.W. and Z.J.C. collected clinical data; J.J.Y., L.X.J., Y.P., and Y.J.H. performed statistical analysis; J.J.Y., Y.J.H., and L.X.J. drafted the manuscript and all the authors reviewed the manuscript.

## Supporting information

Supporting Information

## Data Availability

The data that support the findings of this study are available from the corresponding author upon reasonable request.
