# Supplementary material for: Investigating Novel Therapeutic Approaches for Idiopathic Short Stature: Targeting siRNA and Growth Hormone Delivery to the Growth Plate Using Exosome Nanoparticles
Source: Adv Sci (Weinh). 2024 Apr 19;11(24):2309559. doi: 10.1002/advs.202309559 (PMC11200009; doi:10.1002/advs.202309559)
Supplement: Supplementary file 1 — Supporting Information [file ADVS-11-2309559-s001.pdf]

ADVANCED SCIENCE
Open Access

# Supporting Information

for *Adv. Sci.*, DOI 10.1002/advs.202309559

Investigating Novel Therapeutic Approaches for Idiopathic Short Stature: Targeting siRNA and Growth Hormone Delivery to the Growth Plate Using Exosome Nanoparticles

*Jinghong Yuan, Yameng Wang, Yanzhe Huang, Shengqin Li, Xiaowen Zhang, Zhiwen Wu, Wenrui Zhao, Junchao Zhu, Junqiu Zhang, Guowen Huang, Peng Yu, Xigao Cheng, Xinhui Wang, Xijuan Liu* and Jingyu Jia**

# Supporting Information

## Investigating Novel Therapeutic Approaches for Idiopathic Short Stature: Targeting siRNA and Growth Hormone Delivery to the Growth Plate Using Exosome Nanoparticles

Jinghong Yuan, Yameng Wang, Yanzhe Huang, Shengqin Li, Xiaowen Zhang, Zhiwen Wu, Wenrui Zhao, Junchao Zhu, Junqiu Zhang, Guowen Huang, Peng Yu, Xigao Cheng, Xinhui Wang, Xijuan Liu*, and Jingyu Jia*

**Supplementary Materials**

**1. Molecular docking studies**

The secondary RNA structure prediction of ISSRL and miR-877-3p was executed using the Mfold online tool (http://www. unafold.org/mfold/applications/rna-folding-form.php). Subsequently, RNA Composer was employed to generate 3D-RNA structural models for ISSRL and miR-877-3p. The PDB file of AGO2 was obtained from the Research Collaboratory for Structural Bioinformatics Protein Data Bank (RCSB PDB). To predict the spatial interaction of ISSRL, miR-877-3p, and AGO2, the NPDock website was utilized. Additionally, for visualization purposes, we employed the online tool home-for-researchers (www.home-for-researchers.com).

**2. TEM negative staining**

Took 20 ul of exosome suspensio with a pipet-gun and dropped onto the copper grid with carbon film for 3-5min, and then use filter paper to absorb the excess liquid. Dropped 2% phosphotungstic acid on the copper grid to stain for 1-2min, use filter paper to absorb excess liquid, and dry at room temperature. The cuprum grids are observed under TEM and take images (HITACHI, HT7800/HT7700).

**3. NTA particle size and zeta potential analysis**

Instrument calibration: After the instrument is turned on, dilute the standard solution of 100nm polystyrene microspheres by 250000 times with ultrapure water, and take 1ml of the diluted standard solution for automatic calibration of the instrument; After the automatic calibration of the instrument is completed, dilute the exosomes sample with a clean PBS solution to a suitable concentration, so that the number of particles displayed on the instrument detection interface of the exosomes sample ranges from 50 to 400, preferably around 200; Enter the dilution ratio of the sample in the software interface and observe whether the number of particles displayed at the detection position is close; After confirming that the number of particles displayed at each detection position is very close, click on the software interface in sequence. Measurement, Run Video Acquisition, set the sample name and data saving path in the pop-up interface. Path, select the appropriate SOP, and finally click OK to start testing. The instrument automatically completes the testing process, analyzes data, and then generates a test report, providing information such as particle size, zeta potential, and concentration of the sample.

**4. Load detection**

exosomal RNA extraction was performed using the Exosomal RNA Extraction Kit from NORGEN BIOTEK CORP. Subsequently, reverse transcription was conducted using the PrimeScriptTM RT Reagent Kit with gDNA Eraser (TaKaRa, Japan). For cDNA amplification with si-ISSRL primer sequences (RiboBio), the 2 × Taq PCR Mix kit (TB Green® Premix Ex TaqTM II, TaKaRa, Japan) was employed. A 2% agarose gel was prepared using 1×TBE buffer (Beyotime) and microwaved three times for 1~2 minutes each. Gel staining was achieved with the SYBR Safe DNA gel stain kit (Invitrogen, USA). Agarose gel electrophoresis was carried out using the Bio-Rad agarose horizontal electrophoresis apparatus, and gel imaging was performed using a gel imager from Tanon..

**5. Safranin fast green staining for bone**

Place the paraffin sections in sequence in Environmental Friendly. Dewaxing Transparent Liquid I for 20 min - Environmental Friendly Dewaxing Transparent Liquid II for 20min - Anhydrous ethanol I for 5 min - Anhydrous ethanol II for 5 min - 75% Ethyl alcohol for 5 min, washing with tap water. The slides stained in fast green dye solution for 1-5 minutes, washed away the excess dye solution until the cartilage was colorless, and soaked in 1% hydrochloric acid and alcohol for 10 s.Then washing them with tap water. The slides were stained in saffron dye solution for 1-5 s, and then put into four cylinders of absolute ethanol, where three for rapid dehydration for 5 s, 2 s, 10 s respectively, and kept in the fourth cylinder. The slides were immersed in xylene to transparent for 5 min, sealing with neutral gum. Observed under microscope, and took images for analysis. Cartilage was red or orange-red, and bone formation was green. And some connective tissues were red.

**6. HE staining**

Dewaxing as followed: Xylene I for 20 min; Xylene II for 20 min; 100% ethanol I for 5 min; 100% ethanol II for 5 min; 75% ethanol for 5 min; Rinsing with tap water. Stain sections with Hematoxylin solution for 3-5 min, rinse with tap water. Then treat the section with Hematoxylin Differentiation solution, rinse with tap water. Treat the section with Hematoxylin Scott Tap Bluing, rinse with tap water.85% ethanol for 5 min; 95% ethanol for 5 min; Finally Stain sections with Eosin dye for 5 min. Then, Dehydrate as followed: 100% ethanol I for 5 min; 100% ethanol II for 5 min; 100% ethanol III for 5 min; Xylene I for 5 min; Xylene II for 5 min; Finally seal with neutral gum. Observe with microscope inspection, image acquisition and analysis.

**Supplementary Figures**

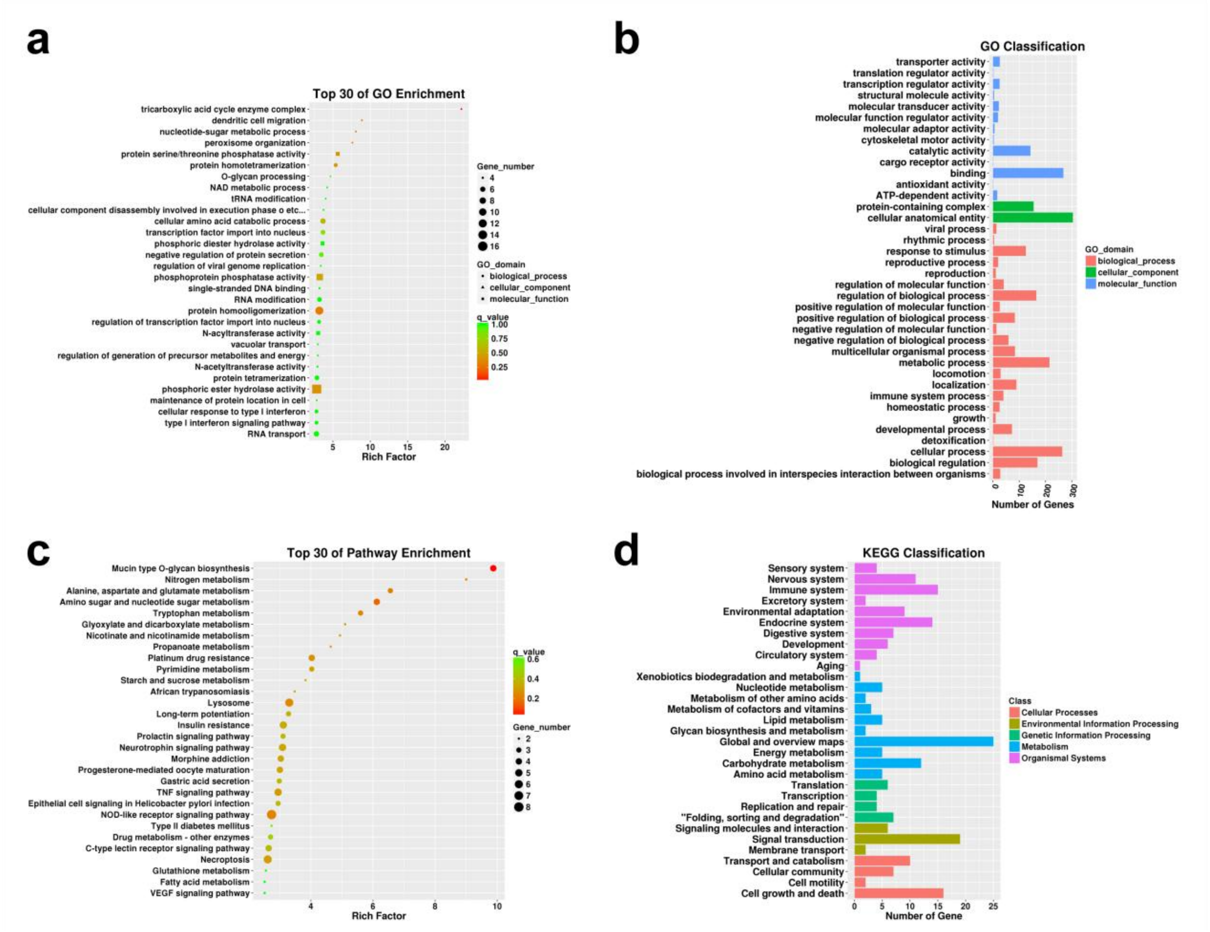


**Figure S1. The Functional Enrichment Analysis of DELs Targeted Genes.** a) A bubble plot of the top 30 of GO enrichment analysis. b) A bar plot of GO classification enrichment analysis. c) A bubble plot of the top 30 of KEGG pathway enrichment analysis. (D) A bar plot of KEGG classification enrichment analysis.

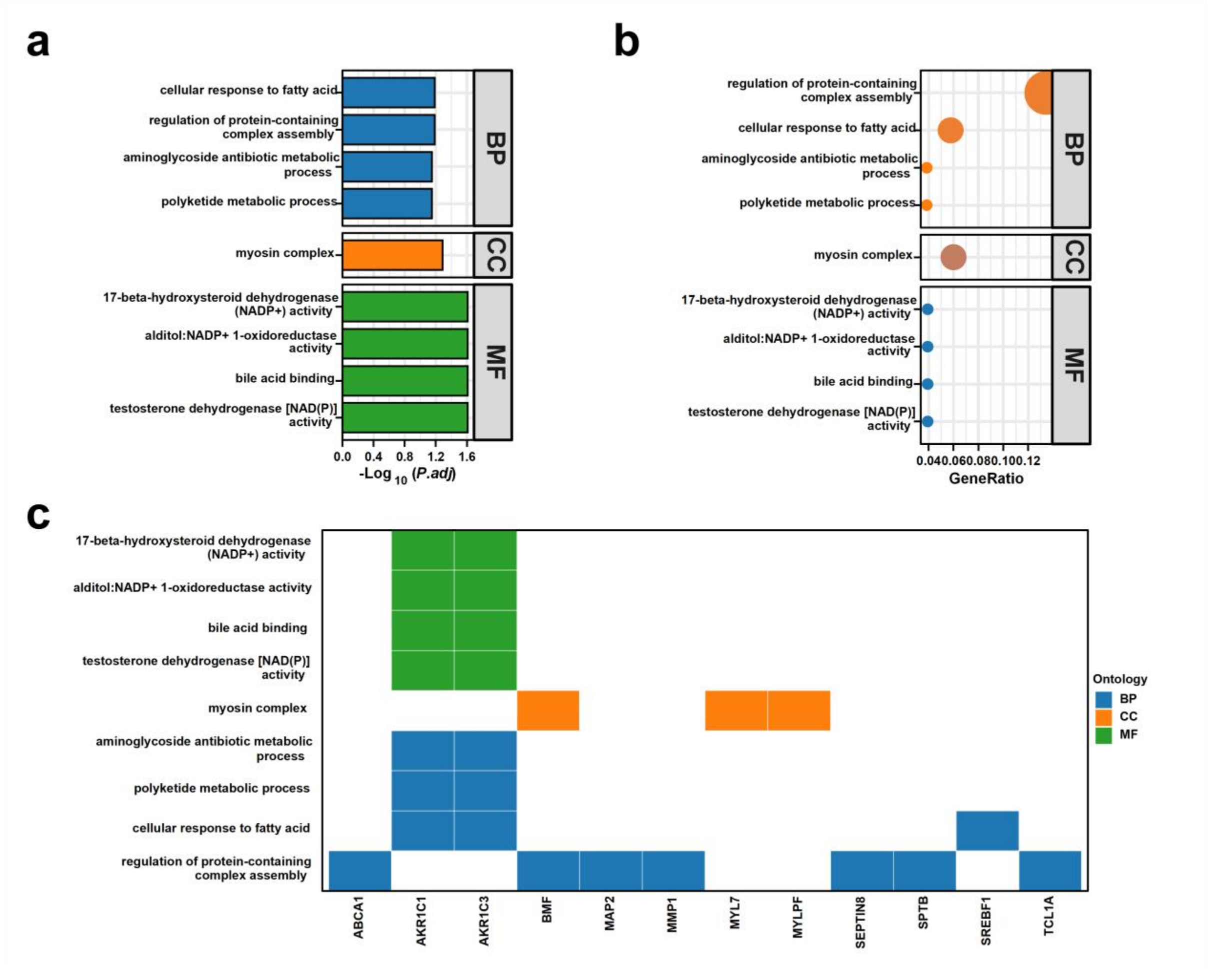


**Figure S2. The Functional Enrichment Analysis of ISSRL-related Genes.** a) A bar plot of GO enrichment analysis of ISSRL-related DEGs. b) A bubble plot of GO enrichment analysis of ISSRL-related DEGs. c) A heatmat plot of GO enrichment analysis of ISSRL-related DEGs.

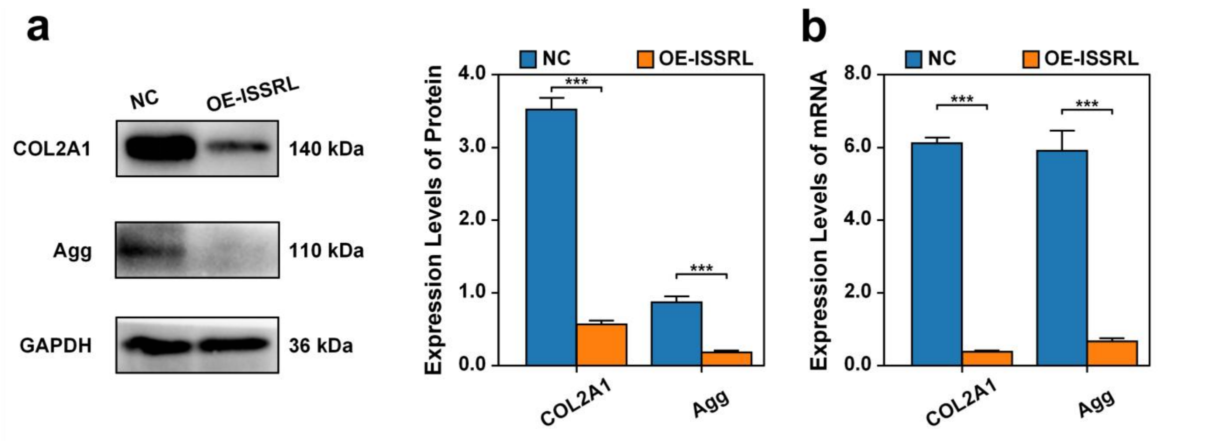


**Figure S3. ISSRL Promoted The ECM Degradation of Human Chondrocytes.** a) Western blot revealed ECM degradation was promoted after overexpression of ISSRL. b) RT-qPCR showed that ECM degradation was promoted after overexpression of ISSRL.

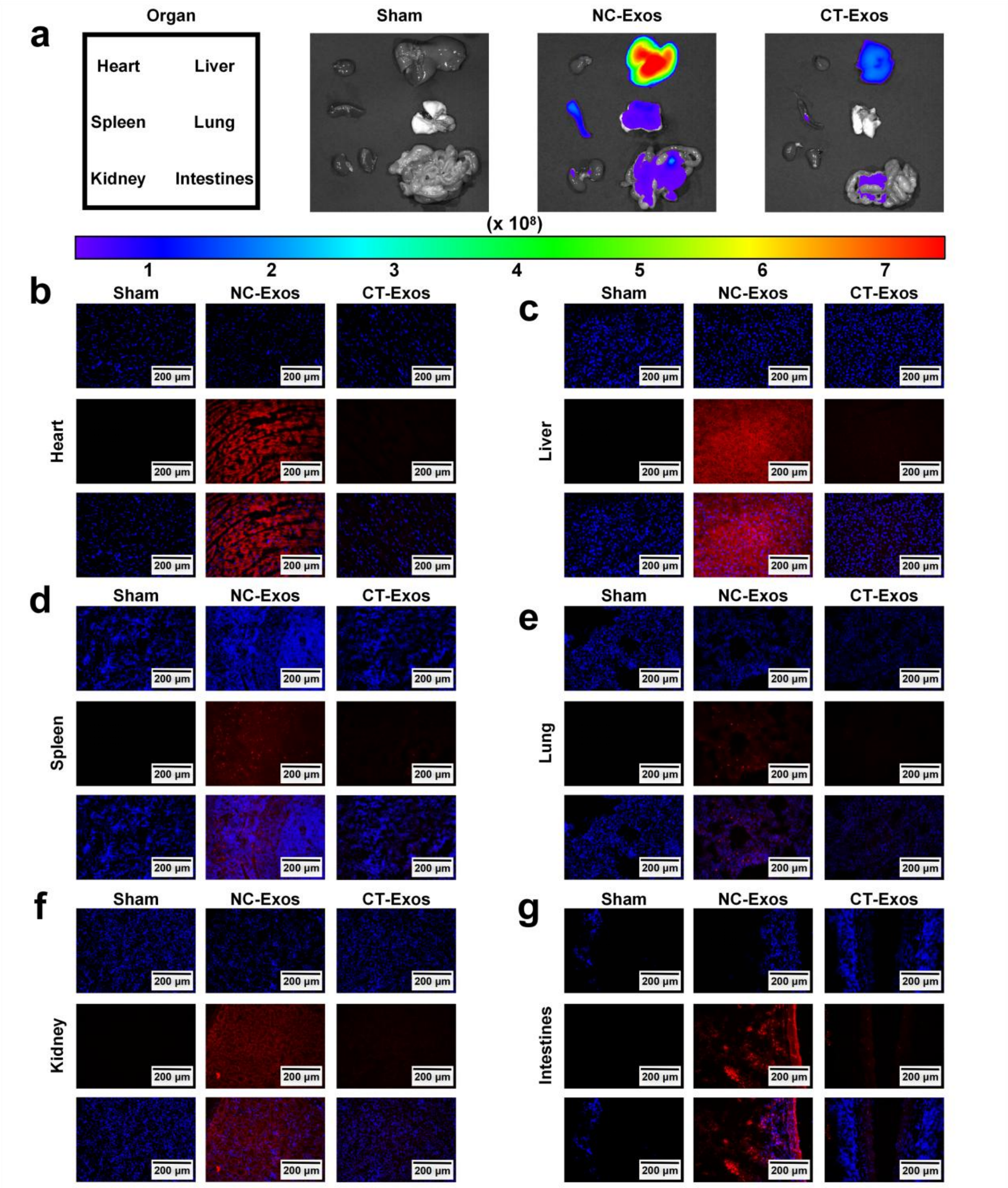


**Figure S4. CT-Exos decreases the UCL of organs compared with NC-Exos.** a) The UCL intensity was noticeably reduced in organs compared between the CT-Exos group and the NC-Exos group. b-g) The Fluorescence imaging of frozen tissue sections was noticeably reduced in organs (heart, liver, spleen, lung, kidney, and intestines) compared between the CT-Exos group and the NC-Exos group.

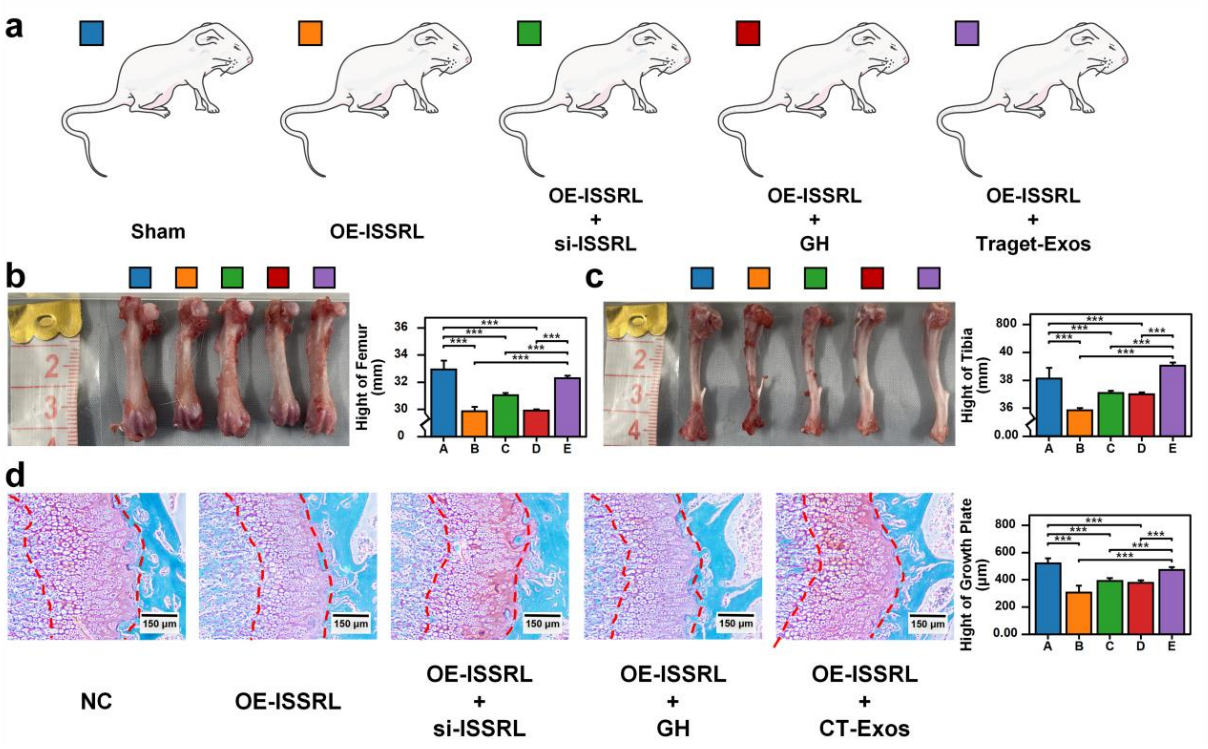


**Figure S5. CT-Exos Significant Recovered The Stature of Rats.** a) Experimental grouping: Group A (Blue): Sham (NC). Group B (Orange): Rats with ISSRL overexpression (OE-ISSRL). Group C (Green): ISSRL-overexpressed rats with concurrent si-ISSRL injection. Group D (Red): ISSRL-overexpressed rats with concurrent GH protein injection. Group E (Purple): ISSRL-overexpressed rats with CT-Exos injection. b) Overexpression of ISSRL resulted in a short phenotype of the femur in rats, CT-Exos significantly recovered the stature of rats. c) Overexpression of ISSRL results in a short phenotype of the tibia in rats, while CT-Exos significantly recovered the stature of rats. d) Safranin O-fast green staining showed that the height of the femoral growth plates in the group B was lesser and CT-Exos significantly recovered the height of the femoral growth plates.

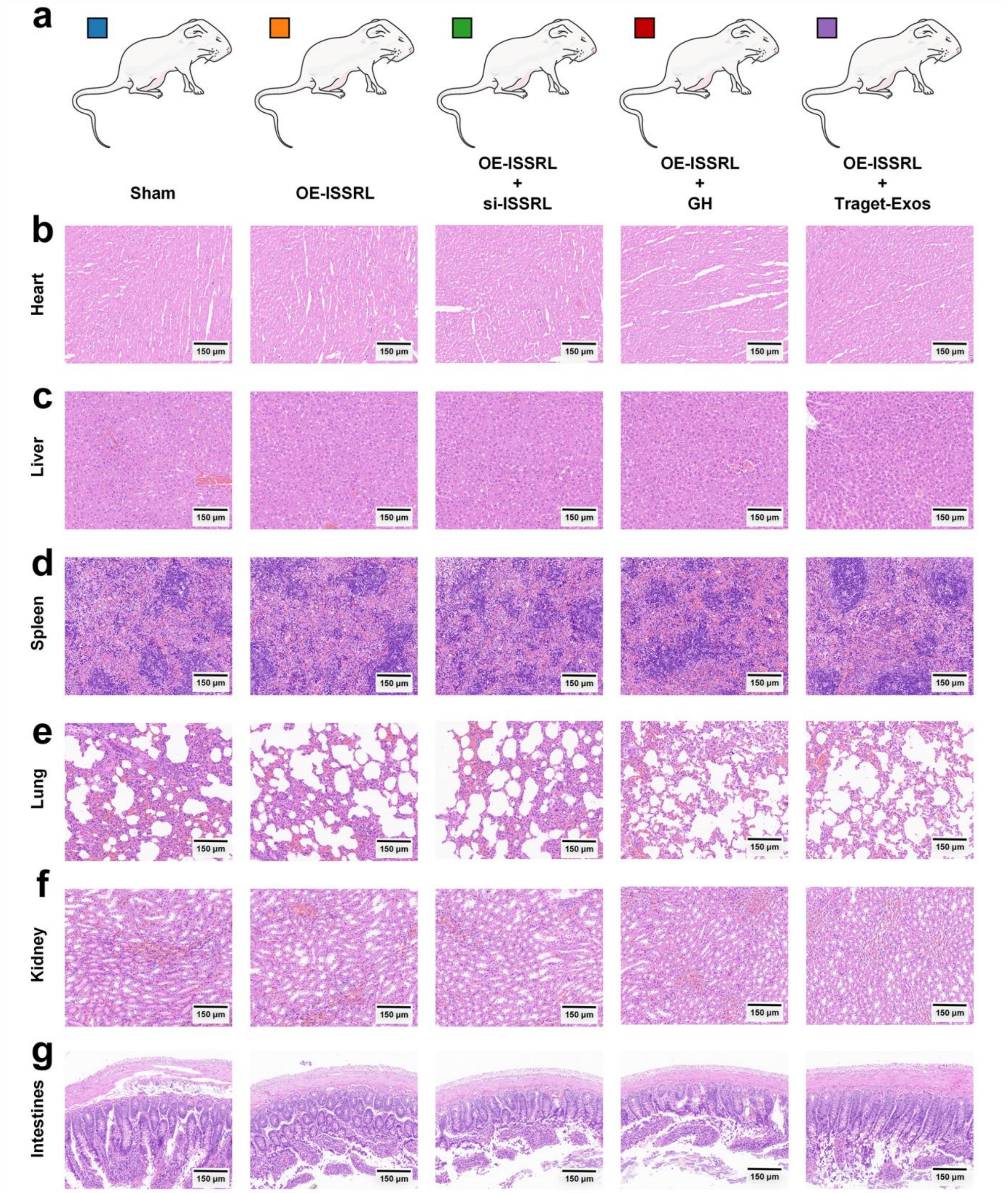


**Figure S6. Safety Evaluation of CT-Exos in Vivo.** a-f) HE Staining Sections of Heart, Liver, Spleen, Lung, Kidney, and Intestine.

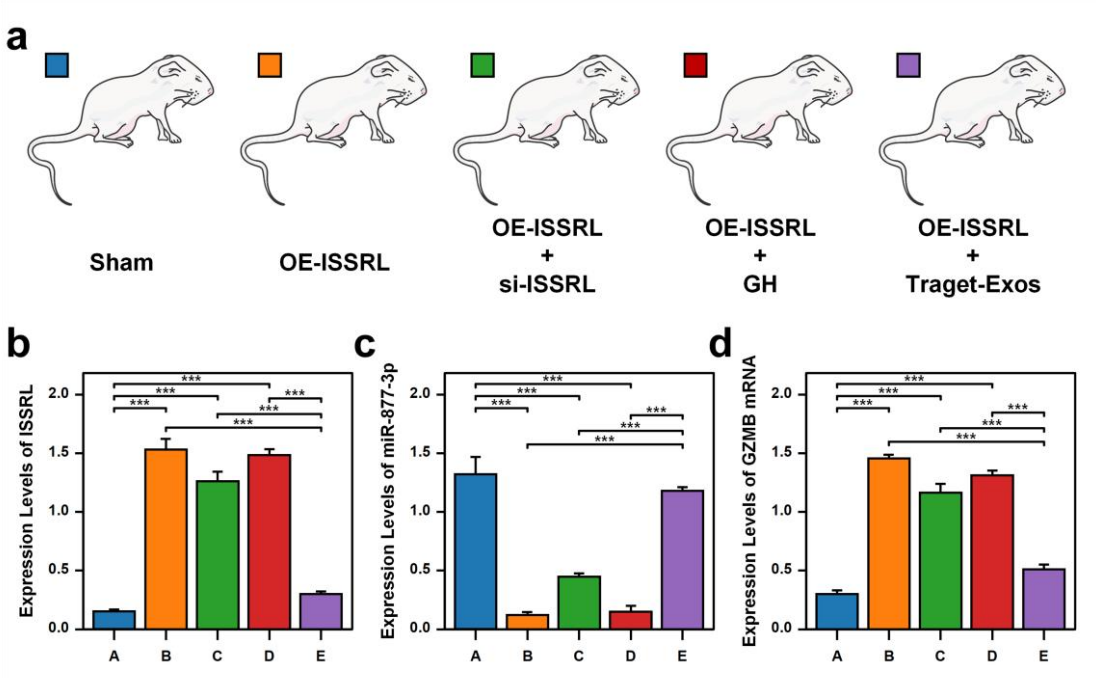


**Figure S7. CT-Exos Regulated The expression of ISSRL, miR-877-3p, and GZMB in growth plate cartilage.** a) Experimental grouping: Group A (Blue): Sham (NC). Group B (Orange): Rats with ISSRL overexpression (OE-ISSRL). Group C (Green): ISSRL-overexpressed rats with concurrent si-ISSRL injection. Group D (Red): ISSRL-overexpressed rats with concurrent GH protein injection. Group E (Purple): ISSRL-overexpressed rats with CT-Exos injection. b) RT-qPCR revealed that ISSRL expression was increased in group B, C and D, and rescued in group E. c) RT-qPCR revealed that miR-877-3p expression was inhibited in group B, C and D, and increased in group E. d) RT-qPCR revealed that GZMB mRNA expression was up-regulated in group B, C and D, and reduced in group E. The data are presented as the mean±SD. n=3. Five groups were compared using ANOVA followed by Tukey's test. *$P<0.05$, **$P<0.01$, ***$P<0.001$ vs. control.

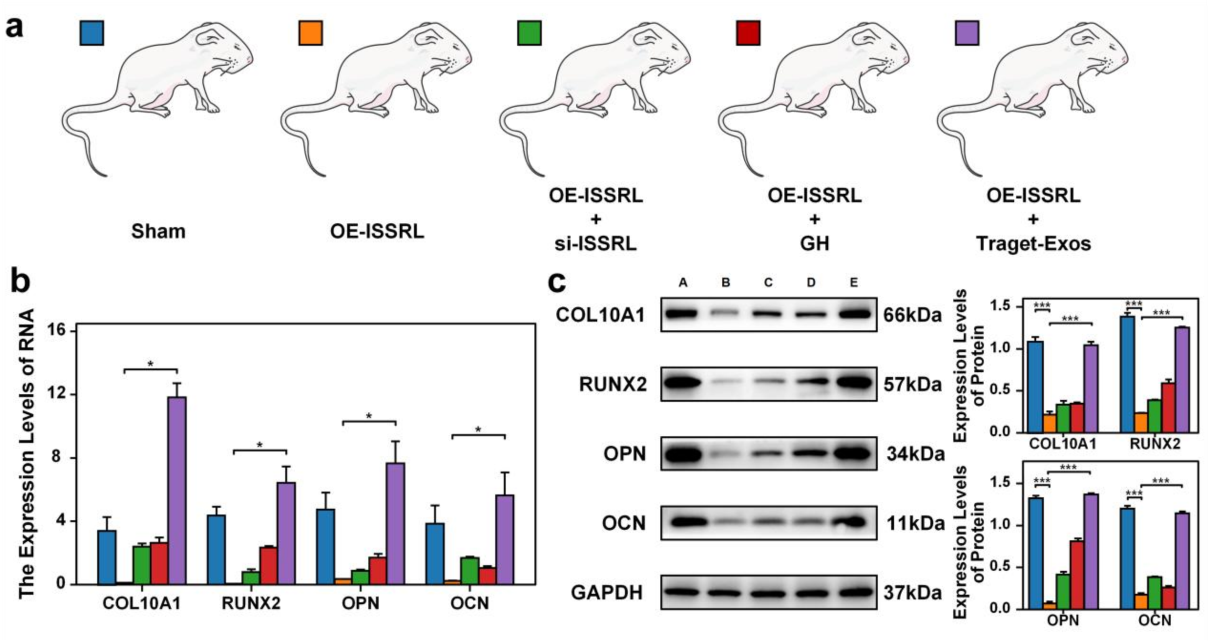


**Figure S8. CT-Exos: Overcoming ISSRL-Mediated Inhibition of Chondrocyte Bone Formation in Rats In Vivo.** a) Experimental grouping: Group A (Blue): Sham (NC). Group B (Orange): Rats with ISSRL overexpression (OE-ISSRL). Group C (Green): ISSRL-overexpressed rats with concurrent si-ISSRL injection. Group D (Red): ISSRL-overexpressed rats with concurrent GH protein injection. Group E (Purple): ISSRL-overexpressed rats with CT-Exos injection. b) RT-qPCR revealed that expression of COL10A1, RUNX2, OPN, and OCN were decreased after overexpression of ISSRL, and the expression levels were increased in group E. c) Western blot showed that the expression of COL10A1, RUNX2, OPN, and OCN were decreased after overexpression of ISSRL, and the expression levels were increased in group E. The data are presented as the mean±SD. n=3. Five groups were compared using ANOVA followed by Tukey's test. *P<0.05, **P<0.01, ***P<0.001 vs. control.

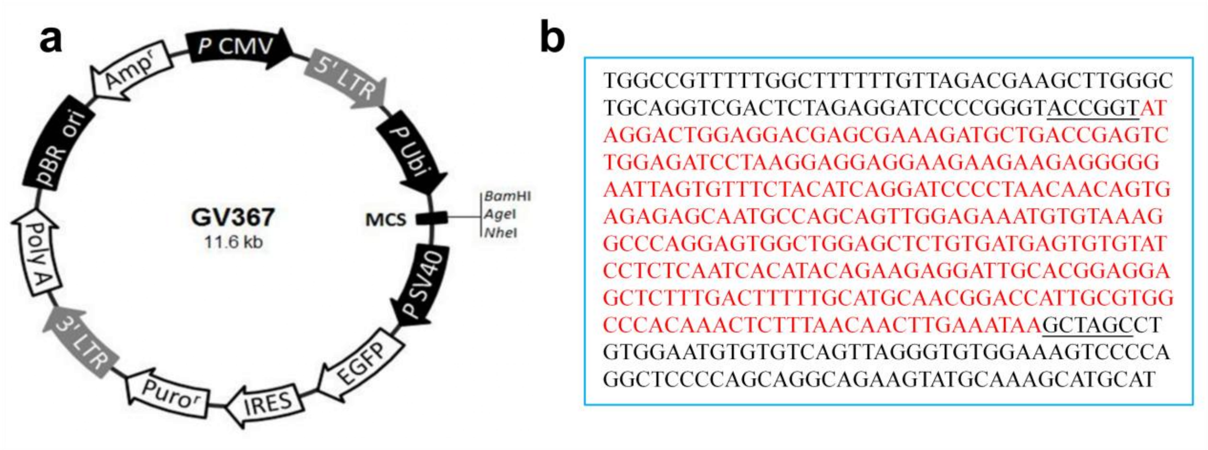


**Figure S9. OE-ISSRL Plasmid.** a) The lncRNA RAB10-2:1 (ISSRL) plasmid (GV 367-Ubi-MCS -SV40-EGFP-IRES-puromycin) was successfully constructed. b) The sequence of lncRNA RAB10-2:1 plasmid or its mutated fragment.

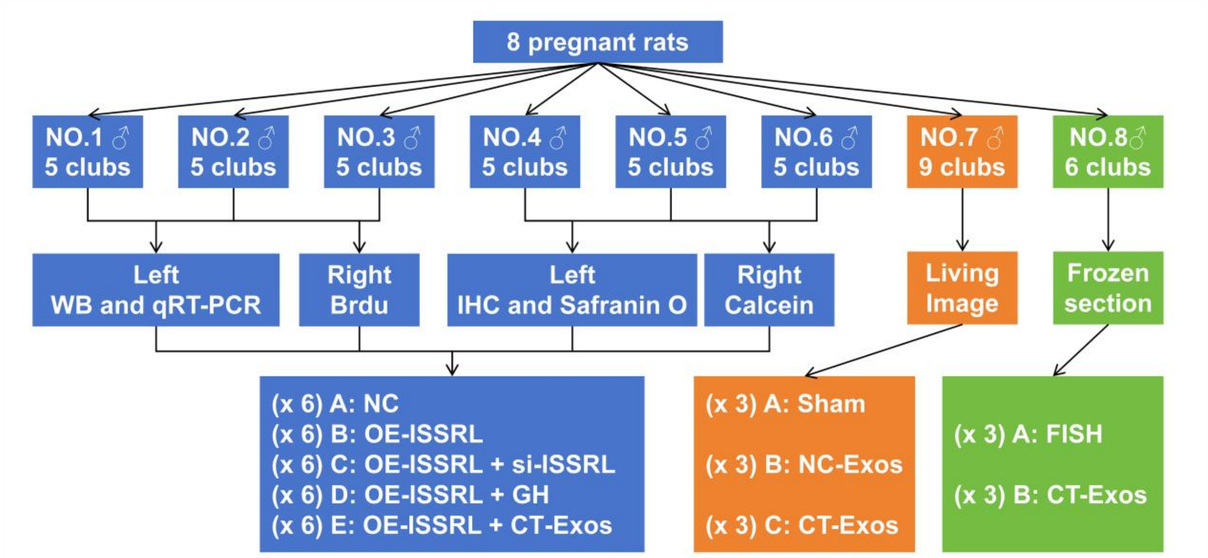


**Figure S10. The Flow Chart Demonstrates The Detailed Grouping Information in Rats.**

## Supplementary Tables

**Table S1.** Primers used for RT-qPCR analysis

| Target ID | | Primer sequence 5'-3' |
|---|---|---|
| **LncRNA-RAB10-2:1 （ISSRL)** | F-primer | TTGTTAAAGAGTTTGTGGG |
| | R-primer | ATAGGACTGGAGGACGAG |
| **GZMB** | F-primer | AGCCGACCCAGCAGTTTATC |
| | R-primer | GTGTGTGAGTGTTTTCCCAGG |
| **GABBR2** | F-primer | GAGTCCACGCCATCTTCAAAAAT |
| | R-primer | TCAGGATACACAGGTCGATCAG |
| **Proteoglycan** | F-primer | CTCCACTGCCTGTGAAGTCACCAC |
| | R-primer | GCACCATGCCTTCTGCTTCCGAG |
| **COL2A1** | F-primer | CGAGGTGACAAAGGAGAAGC |
| | R-primer | CTGGTTGTTCAGCGACTTGA |
| **RUNX2** | F-primer | ACTTCCTGTGCTCCGTGCTG |
| | R-primer | TCGTTGAACCTGGCTACTTGG |
| **COL10** | F-primer | GCAGCATTACGACCCAAGAT |
| | R-primer | CATGATTGAACTCCCTGAAG |
| **OPN** | F-primer | CCAGCCAAGGACCAACTACA |
| | R-primer | AGTGTTTGCTGTAATGCGCC |
| **OCN** | F-primer | GCACCACCGTTTAGGGCAT |
| | R-primer | CGTTCCTCATCTGGACTTTATTTG |
| **GAPDH** | F-primer | GGAGCGAGATCCCTCCAAAAT |
| | R-primer | GGCTGTTGTCATACTTCTCATGG |
| **U6** | F-primer | CGCTTCGGCAGCACATATAC |
| | R-primer | AAATATGGAACGCTTCACGA |
| **hsa-miR-6720-5p** | F-primer | TGCGCTTCCAGCCCTGGTAGGCG |
| | Loop-primer | TCGTATCCAGTGCAGGGTCCGAGGTA TTCGCACTGGATACGACCGCGGCGC |
| **hsa-miR-6824-5p** | F-primer | TGCGCGTAGGGGAGGTTGGGCC |
| | Loop-primer | GTCGTATCCAGTGCAGGGTCCGAGGT ATTCGCACTGGATACGACTCCCTGGC |
| **hsa-miR-877-3p** | F-primer | TGCGCTCCTCTTCTCCCTCCT |
| | Loop-primer | GTCGTATCCAGTGCAGGGTCCGAGGT ATTCGGATACGACCTGGGAGG |
| **hsa-miR-766-3p** | F-primer | TGCGCACTCCAGCCCCACAGCC |
| | Loop-primer | GTCGTATCCAGTGCAGGGTCCGAGGT ATTCGCACTGGATACGACGCTGAGGC |
| **hsa-miR-939-3p** | F-primer | TGCGCCCCTGGGCCTCTGCTC |
| | Loop-primer | GTCGTATCCAGTGCAGGGTCCGAGGT ATTCGCACTGGATACGACCTGGGGAG |
| **hsa-miR-5193** | F-primer | TGCGCTCCTCCTCTACCTCATC |
| | Loop-primer | GTCGTATCCAGTGCAGGGTCCGAGGT ATTCGCACTGGATACGACACTGGGAT |
| **hsa-miR-4691-5p** | F-primer | TGCGCGTCCTCCAGGCCATGAGC |
| | Loop-primer | GTCGTATCCAGTGCAGGGTCCGAGGT ATTCGCACTGGATACGACCCGCAGCT |

| | | |
|---|---|---|
| **hsa-miR-4322** | F-primer | TGCGCCTGTGGGCTCAGCGCG |
| | Loop-primer | GTCGTATCCAGTGCAGGGTCCGAGGT ATTCGCACTGGATACGACCCCCACGC |
| **hsa-miR-1256** | F-primer | TGCGCAGGCATTGACTTCTCAC |
| | Loop-primer | GTCGTATCCAGTGCAGGGTCCGAGGT ATTCGCACTGGATACGACAGCTAGTG |
| **hsa-miR-1343-3p** | F-primer | TGCGCCTCCTGGGGCCCGCACT |
| | Loop-primer | GTCGTATCCAGTGCAGGGTCCGAGGT ATTCGCACTGGATACGACGCGAGAGT |
| **hsa-miR-4691-3p** | F-primer | TGCGCCCAGCCACGGACTGAGAG |
| | Loop-primer | GTCGTATCCAGTGCAGGGTCCGAGGT ATTCGCACTGGATACGACATGCACTC |
| **hsa-miR-6875-3p** | F-primer | TGCGCATTCTTCCTGCCCTGGC |
| | Loop-primer | GTCGTATCCAGTGCAGGGTCCGAGGT ATTCGCACTGGATACGACATGGAGCC |
| **hsa-miR-3605-3p** | F-primer | TGCGCCCTCCGTGTTACCTGTCC |
| | Loop-primer | GTCGTATCCAGTGCAGGGTCCGAGGT ATTCGCACTGGATACGACCTAGAGGA |
| **hsa-miR-4713-3p** | F-primer | TGCGCTGGGATCCAGACAGTGG |
| | Loop-primer | GTCGTATCCAGTGCAGGGTCCGAGGT ATTCGCACTGGATACGACCCCAGAGC |
